# Blood-activating, depression-relieving formula alleviates post-stroke depression: mechanistic insights from network pharmacology and microglial validation

**DOI:** 10.3389/fneur.2026.1780535

**Published:** 2026-07-02

**Authors:** Na Zhao, Lumi Zhang, Wei Li, Yiru Wang, Zhengyu Zhu, Zhimin Wu

**Affiliations:** Department of Neurology, Wenzhou TCM Hospital of Zhejiang Chinese Medical University, Zhejiang, China

**Keywords:** EGFR-STAT3, microglia, network pharmacology, Pi3k-akt, post-stroke depression, traditional Chinese medicine

## Abstract

**Introduction:**

Post-stroke depression (PSD) is common and disabling, yet mechanism-based, multi-target therapies that jointly curb neuroinflammation and support cell survival are scarce. We evaluated a Blood-Activating, Depression-Relieving (BADR) herbal formula for effects on PSD-relevant molecular hubs and microglial phenotypes.

**Methods:**

BADR constituents from Traditional Chinese Medicine Systems Pharmacology Database and Analysis Platform were standardised and mapped to human protein targets. Target-disease interaction networks were assembled in Search Tool for the Retrieval of Interacting Genes/Proteins, clustered with Molecular Complex Detection, and functionally annotated via Kyoto Encyclopedia of Genes and Genomes Orthology-Based Annotation System (KEGG/GO). For experimental validation, BV2 microglia were activated with lipopolysaccharide (LPS; 24 h) and co-treated with BADR within a pre-established non-cytotoxic range; dexamethasone (1 μM) served as comparator. Outcomes included cytokines (IL-1β, TNF-*α*, IL-6; enzyme-linked immunosorbent assay), expression of selected nodes (EGFR, STAT3, JUN, PIK3CA, BCL2; quantitative real-time polymerase chain reaction/western blot), viability (Cell Counting Kit-8), and apoptosis (flow cytometry).

**Results:**

Network analysis highlighted two dense modules enriched for PI3K-AKT, JAK–STAT, and neuroactive-ligand signaling. Hubs included EGFR, AKT1, STAT3, JUN, PIK3CA, and BCL2, with EGFR, STAT3, PIK3CA, JUN, and BCL2 prioritised for cellular validation based on topology, pathway relevance, and compound-target connectivity. In BV2 cells, BADR attenuated LPS-induced IL-1β, TNF-*α*, and IL-6 surges, improved viability, and reduced total apoptosis, with directionally comparable effects to dexamethasone. Mechanistically, BADR down-regulated EGFR/JUN/STAT3/PIK3CA and restored BCL2 at transcript and protein levels.

**Conclusion:**

By converging network-level predictions with microglial phenotyping, the formula exerts coordinated anti-inflammatory and pro-survival effects centred on the EGFR-STAT3-PI3K nodes in a PSD-relevant context. These data provide a mechanistic rationale for further phosphorylation-level and *in vivo* validation toward multi-target PSD therapeutics.

## Introduction

Post-stroke depression (PSD) is a common and debilitating neuropsychiatric consequence of cerebrovascular injury. It impairs functional recovery, increases the risk of complications, and reduces the quality of life for patients and their caregivers (1, 2). Research by SHI et al. in 2023 indicated that PSD pathogenesis reflects a complex convergence of ischaemia-triggered neuroinflammation, disrupted neuromodulatory circuits, metabolic stress, vascular dysfunction, and psychosocial burden. This yields a heterogeneous clinical phenotype that is difficult to treat with single-target drugs (3). Current management relies on modulating monoaminergic transmission symptomatically and providing supportive rehabilitation. However, the delayed onset of action, incomplete remission, drug–drug interactions with secondary prevention regimens and vulnerability to adverse events limit its effectiveness in the real world (4). A recent study shows that, mechanistically, post-ischemic tissue is characterised by microglial activation, cytokine amplification, oxidative and hypoxic stress, and maladaptive plasticity within corticolimbic networks that govern mood and cognition (5). These processes are orchestrated by receptor-kinase and transcriptional crosstalk programmes, intersecting with neurotransmitter systems that regulate affect and motivation. Therefore, the therapeutic bottleneck is not simply one of insufficient potency at a single molecular node, but of fragmentation between inflammatory control, cellular survival, and synaptic regulation. Multi-target strategies that can coordinate upstream receptor signaling, intracellular effectors, and secretory outputs are urgently needed. Against this background, we set out to mechanistically link a traditional Blood-Activating, Depression-Relieving (BADR) formula, which has been used empirically for a long time in neuropsychiatric contexts, to PSD-relevant molecular networks and microglial phenotypes. Our aim was to rationalise its polypharmacology in a modern systems framework.

Among the cellular processes linking post-stroke injury to depressive phenotypes, microglia-driven neuroinflammation provides a particularly relevant entry point for mechanistic investigation. Ischaemic injury mobilises innate immune surveillance, and microglia rapidly transition to activated states that reshape the perivascular and synaptic environments (6). Within these cells, receptor tyrosine kinases, such as the epidermal growth factor receptor (EGFR), interact with phosphoinositide-3 kinase catalytic subunits, including PIK3CA, to activate AKT and downstream survival checkpoints. Meanwhile, parallel channels converge on transcription factors, such as STAT3 and JUN (7–9). Activated microglia also integrate extracellular cues from cytokines and chemokines, modulating the production of prototypical inflammatory mediators such as interleukin (IL)-1β, IL-6, and tumour necrosis factor (TNF-*α*) through tightly regulated signaling cascades. Hypoxia-inducible pathways, which are mediated by HIF-1α, provide an additional layer of control in post-ischemic tissue. These pathways influence angiogenic and metabolic adaptation, as well as tuning inflammatory tone (10). Importantly, these axes do not operate in isolation. EGFR-initiated inputs can feed into PI3K-AKT cascades that regulate apoptosis and mitochondrial integrity via BCL2 family checkpoints. Meanwhile, STAT3 integrates cytokine receptor signaling to sculpt transcriptional programmes that influence both inflammatory thresholds and survival (11). These interconnected inflammatory and survival pathways provide a biologically grounded target space for network pharmacology-based prioritisation and subsequent cellular validation.

In traditional Chinese medicine theory, post-stroke depression is commonly associated with “qi” stagnation, blood stasis, and dysregulation of “shen” after stroke. The Blood-Activating, Depression-Relieving formula (BADR) used in this study corresponds to the therapeutic principle of activating blood circulation and relieving depressive constraint, and related formulas have also been described in the literature as Jieyu Huoxue Decoction or Huoxue Jieyu Decoction. Previous clinical observations have suggested that such blood-activating and depression-relieving prescriptions may alleviate depression after cerebral infarction and depression after craniotomy, supporting their empirical use for depressive symptoms occurring after brain injury (12, 13). The BADR formula used in this study consists of Astragali Radix, Bupleuri Radix, Chuanxiong Rhizoma, Angelicae Sinensis Radix, Persicae Semen, Carthami Flos, Paeoniae Radix Rubra, Paeoniae Radix Alba, Curcumae Radix, Pheretima, Aurantii Fructus Immaturus, Acori Tatarinowii Rhizoma. These herbs provide a chemically diverse mixture of bioactive constituents, including flavonoids, saponins, phenolic acids, phthalides, monoterpene glycosides, curcuminoids, and other small-molecule components that may contribute to the multi-target pharmacological profile of the formula. This chemical diversity suggests that BADR may exert distributed effects across multiple molecular nodes rather than acting on a single target. Network pharmacology therefore provides a suitable systematic strategy for translating this polypharmacological feature into testable hypotheses by mapping curated constituents to predicted protein targets, intersecting these targets with disease-associated genes, and prioritising hub proteins and pathways within protein–protein interaction networks (14, 15). In traditional Chinese medicine research, this approach is commonly combined with functional enrichment analysis to identify biologically plausible pathways and candidate target-ligand interactions (16, 17). In this context, the chemical diversity of BADR provides a rational basis for investigating its potential engagement of receptor tyrosine kinase inputs centred on EGFR, intracellular PI3K-AKT survival effectors, JAK–STAT transcriptional control, PTGS2-linked eicosanoid biosynthesis, and neuroactive-ligand interfaces pertinent to PSD, thereby motivating the subsequent network-pharmacology mapping and BV2 microglial assays (18–24).

It is crucial that computational inferences are based on tractable cellular systems that capture the neuroinflammatory context of PSD. LPS-stimulated BV2 microglia are commonly used as a tractable *in vitro* system for quantifying cytokine release, transcriptional activity at prioritised nodes, and downstream apoptosis/viability changes under microglial inflammatory stress (25, 26). Because microglia-driven neuroinflammation and IL-1β, IL-6, and TNF-*α* release are well-recognised contributors to PSD pathophysiology (5, 27), and because BV2 microglia have been used in PSD-related studies to interrogate microglial inflammatory signaling (28), LPS-activated BV2 cells were used here to model a PSD-relevant inflammatory component rather than the full clinical syndrome of PSD. Within this framework, the aim of this study was to integrate a network-pharmacology map of a BADR formula with microglial assays that query cytokine outputs, prioritised receptor-kinase and transcriptional nodes, and survival phenotypes in a PSD-relevant context. By aligning predictions of constituents and targets, disease-anchored interaction modules, and cell-based readouts, this study seeks to provide a mechanistic rationale for the modulation of multiple targets in order to influence neuroinflammation and cell survival in PSD.

## Materials and methods

### Reagents, consumables

LPS from *E. coli* O111: B4 (MedChemExpress, NJ, USA) was prepared at a concentration of 1 mg/mL in phosphate-buffered saline (PBS). The dexamethasone (DXMS) benchmark was 1 μM. Other reagents and consumables included PBS, radioimmunoprecipitation assay, Tris-buffered saline, Tween-20, Triton X-100, SDS, non-fat milk, a bicinchoninic acid kit, PVDF membranes, a Cell Counting Kit-8 (CCK-8) kit, an Annexin V-fluorescein isothiocyanate (FITC) apoptosis kit, and 4′,6-diamidino-2-phenylindole (DAPI), all from Beyotime (Shanghai, China). ECL A/B from NCM Biotech (Suzhou, China).

### Herbal formula preparation

Crude herbs composing the formula were authenticated to species level and comprised Astragali Radix *(Astragalus membranaceus (Fisch.) Bunge root [Fabaceae])*, Bupleuri Radix *(B. scorzonerifolium Willd. root [Apiaceae])*, Chuanxiong Rhizoma *(Ligusticum chuanxiong Hort. rhizome [Apiaceae])*, Angelicae Sinensis Radix *(Angelica sinensis (Oliv.) Diels root [Apiaceae])*, Persicae Semen, charred (chao) *(P. davidiana (Carr.) Franch seed [Rosaceae])*, Carthami Flos *(Carthamus tinctorius L. flower [Asteraceae])*, Paeoniae Radix Rubra *(Paeonia lactiflora Pall. raw root [Paeoniaceae])*, Paeoniae Radix Alba *(Paeonia lactiflora Pall. processed root [Paeoniaceae])*, Curcumae Radix *(Curcuma wenyujin Y. H. Chen & C. Ling tuber [Zingiberaceae])*, Pheretima *(Pheretima aspergillum (E. Perrier) body [Megascolecidae])*, Aurantii Fructus Immaturus *(Citrus × aurantium L. immature fruit [Rutaceae])*, and Acori Tatarinowii Rhizoma *(Acorus tatarinowii Schott rhizome [Acoraceae])*, and all of them were purchased from Kangmei Pharmaceutical Co., LTD (Guangdong, China). Authenticated herbs were weighed according to the clinical ratio, decocted in water (1:10, w/v) for 2 × 60 min at 95–100 °C, filtered (100 μm), combined, concentrated under reduced pressure, and lyophilized to a dry powder. The powder was stored desiccated at −20 °C and reconstituted in complete medium immediately before use; reconstitutes were passed through a 0.22 μm sterile filter with pH adjusted to 7.2 and osmolality to 280–320 mOsm/kg. For cell treatment, dose ranges were established by preliminary CCK-8 tolerability testing (24 h exposure), and all working solutions were vehicle-matched (≤0.1% v/v).

### Compound standardisation and target prediction

Putative bioactive constituents were retrieved from the Traditional Chinese Medicine Systems Pharmacology Database and Analysis Platform (TCMSP) for each herb. All database queries were performed and compound entries were first standardised by compound name, canonical SMILES, InChIKey, and PubChem CID to minimise redundancy across herbs and databases. Duplicate compounds were removed primarily according to identical InChIKey values, and compounds lacking usable structural identifiers were manually checked before exclusion. Targets were predicted using SwissTargetPrediction and BATMAN-TCM for *Homo sapiens* only, and only targets with valid human gene annotations were retained for downstream analysis. For SwissTargetPrediction, the species was restricted to human, and candidate targets were recorded together with the platform-derived probability values. For BATMAN-TCM, targets were retained according to the default or pre-specified score threshold used in the platform at the time of retrieval, and the score information was preserved for traceability. The results were collated into a compound-target table containing UniProt accessions, HUGO Gene Nomenclature Committee (HGNC) gene symbols, target class, evidence/source, and cross-references (e.g., ChEMBL). Target names from different sources were normalised to official HGNC gene symbols and UniProt-reviewed entries, and duplicated target records from multiple platforms were merged while preserving the original source information and prediction scores. The final mapping used only human proteins.

### Data collection and network construction

PSD-related genes were compiled by querying DisGeNET, GeneCards, and the Therapeutic Target Database (TTD) using the terms ‘PSD’, ‘stroke depression’ and related synonyms. The results were deduplicated based on the HGNC symbol and the UniProt ID. Genes retrieved from multiple disease databases were pooled and standardised to official HGNC symbols before intersection analysis. Compound-mapped human targets were then intersected with the PSD-related gene set to define a disease-anchored candidate target pool. A multipartite network (compound-target-disease) was constructed in Cytoscape (version 3.8) with node/edge attributes (compound class, target class, source counts, and evidence). Degree, betweenness centrality, and neighbourhood connectivity were computed using NetworkAnalyzer. To improve the transparency of core target selection, topological parameters, including degree and betweenness centrality, were extracted and ranked for the intersecting targets, and these metrics were used together with pathway relevance to prioritise hub genes for subsequent validation. A Sankey diagram (compound-target-disease) was generated from the same table to visualise many-to-many pharmacology.

### PPI modelling and module detection

The intersection genes were projected onto Search Tool for the Retrieval of Interacting Genes/Proteins (STRING, human; version 11; minimum interaction score 0.7; active interaction sources: experiments, curated databases, co-expression and text mining). The resulting protein–protein interaction (PPI) graph was then imported into Cytoscape and clustered using Molecular Complex Detection (MCODE) plugin with the following parameters: degree cutoff = 2, node score cutoff = 0.2, K-core = 2 and max depth = 100. Cluster membership, seed scores, and intra-cluster densities were then exported for subsequent analysis. In parallel, network topology analysis was performed on the full PPI network to identify hub nodes with high centrality, and these ranked hub genes were considered together with module membership and biological relevance when selecting EGFR, STAT3, PIK3CA, JUN, and BCL2 for downstream experimental assessment.

### Functional enrichment analysis

Pathway and ontology enrichment were performed in KEGG Orthology-Based Annotation System (v3) for (i) the full intersection set and (ii) each MCODE cluster. KEGG (Kyoto Encyclopedia of Genes; build/version recorded) and GO (Genomes Orthology-Based Annotation System; BP (biological process)/MF (molecular function)/CC (cellular component); release date recorded) were queried using the human genome as the background reference. Over-representation testing employed hypergeometric/Fisher’s exact tests with Benjamini-Hochberg false discovery rate (FDR) correction (q < 0.05 was considered significant). The enrichment outputs (term ID, description, gene overlap, raw *p* value, FDR q value and odds ratio) were visualised in R (version 4.0+) using dot plots and chord/river plots (e.g., clusterProfiler and ggplot2).

### LC–MS/MS-based chemical characterisation of BADR extract

Three independently prepared BADR extract samples were analysed by LC–MS/MS (liquid chromatography–tandem mass spectrometry). Lyophilized BADR powder (50 mg) was mixed with 1 mL of 70% methanol–water, vortexed for 30 s, sonicated in an ice bath for 30 min, and centrifuged at 13,000 × g for 10 min. The supernatant was collected, dried, and stored at −80 °C. Before analysis, the dried extract was reconstituted in 200 μL of 50% methanol–water, vortexed for 1 min, sonicated for 15 min, centrifuged at 13,000 × g at 4 °C for 30 min, and filtered through a 0.22 μm membrane. Chromatographic separation was performed on a Waters ACQUITY ultra-performance liquid chromatography system with an ACQUITY UPLC BEH C18 column (1.7 μm, 2.1 mm × 100 mm). The column temperature was 40 °C, the flow rate was 0.3 mL/min, the injection volume was 10 μL, and the autosampler temperature was 10 °*C. mobile* phase A was water containing 0.1% formic acid, and mobile phase B was acetonitrile. The gradient was 0–1 min, 95% A; 1–4 min, 95% A to 70% A; 4–11 min, 70% A to 50% A; 11–13 min, 50% A to 20% A; 13–14 min, 20% A to 0% A; 14–16 min, 0% A; 16–16.1 min, 0% A to 95% A; and 16.1–20 min, 95% A. Mass spectrometric data were acquired on an AB Sciex TripleTOF 5,600 system in both positive and negative ion modes. The spray voltage was +5,000 V in positive mode and −4,500 V in negative mode. Gas 1 and Gas 2 were set at 60 psi, curtain gas at 30 psi, source temperature at 600 °C, and declustering potential at ±60 V. MS data were collected over an m/z range of 60–1,200, and MS/MS data were collected over an m/z range of 25–1,200. Collision energy was set at 10 eV for MS and 35 ± 15 eV for MS/MS. Data-dependent acquisition mode was used. Raw data were processed using MS-DIAL for peak alignment, retention-time correction, and peak-area extraction. Metabolite annotation was performed with first-order mass tolerance < 0.01 Da, second-order mass tolerance < 0.02 Da, and MS/MS matching score > 70%. Total ion chromatograms and base peak chromatograms were used for chemical profiling of BADR extract. Batch-level fingerprint consistency was assessed across the three independently prepared BADR samples.

### Cell culture and treatment

BV2 murine microglia-like cells (Xiamen Yimo Biotechnology, Xiamen, China) were cultured in high-glucose DMEM (Gibco, Thermo Fisher, MA, USA), supplemented with foetal bovine serum (FSD500, ExCell Bio, Shanghai, China), at 37 °C in 5% CO_2_. BV2 cells were seeded at the indicated densities (see the relevant sections for each assay) and allowed to attach overnight. For inflammatory activation, the cells were exposed to LPS (0.1–10 μg/mL) for 24 h. This LPS-stimulated BV2 system was used as a PSD-relevant microglial inflammatory model to assess cytokine release and apoptosis-associated cellular injury, given the recognised contribution of microglia-driven neuroinflammation, including IL-1β, TNF-*α*, and IL-6 signaling, to PSD pathophysiology (5, 27). Apoptosis-associated readouts were included to reflect inflammation-related microglial stress and survival changes rather than to model the full complexity of PSD. For the intervention, the BADR formula was added 1 h before the LPS (pretreatment) and was maintained throughout the 24-h stimulation period, unless otherwise stated. DXMS (1 μM) served as a positive control. Vehicle volumes were equalised across groups. Endpoint harvesting adhered to fixed time windows to minimise temporal variability. BV2 morphology was documented under phase contrast (10x objectives) after 24 h treatment. Representative fields were imaged at fixed exposure settings. For rescue experiments, BV2 cells were assigned to five groups: Control, LPS, LPS + BADR, LPS + EGF (epidermal growth factor), and LPS + BADR+EGF. BADR was added 1 h before LPS stimulation. EGF (2028-EG, R&D Systems, MN, USA) was used as an EGFR agonist and was added at 50 ng/mL for 30 min before LPS treatment. Cells and culture supernatants were collected for western blotting and enzyme-linked immunosorbent assay (ELISA) after the indicated treatments.

### Cell viability assay

Cells were seeded in 96-well plates at a density of 6 × 10^3^ cells per well in 100 μL of medium. After 24 h, 10 μL of CCK-8 reagent was added to each well, and the plates were incubated for 1 h at 37 °C, protected from light. Absorbance was read at 450 nm with a 650–570 nm reference. The blank-corrected signals were normalised to the vehicle control and expressed as a percentage of viability.

### Elisa

Culture supernatants were collected at 24 h, cleared (300 × g, 5 min), and assayed for IL-1β, TNF-*α*, and IL-6 using sandwich ELISA kits according to the manufacturer’s instructions. Standard curves (four-parameter logistic fits) were generated in duplicate. Absorbance was measured at 450 nm with correction at 570 nm.

### Quantitative real-time polymerase chain reaction (qRT-PCR)

Total RNA was isolated from BV2 cells 24 h after the indicated treatments using a silica-membrane spin kit with on-column gDNA removal. The cells were lysed directly in Buffer RL, clarified through a genomic DNA filter, and bound to RNA columns in the presence of ethanol. The RNA was then eluted twice with RNase-free water. For cDNA synthesis, 500 ng of total RNA was first incubated with gDNA wiper mix and then reverse transcribed in a 20 μL system containing Hiscript III enzyme, oligo(dT)20VN, and random hexamers. This yielded cDNA following incubation at 37 °C and brief heat inactivation. qPCR reactions were amplified on a CFX96 instrument using the following programme: 95 °C for 30 s, followed by 40 cycles of 95 °C for 10 s and 60 °C for 10 s.

The target genes were IL-1β, TNF-*α*, IL-6, EGFR, JUN, STAT3, and PIK3CA, with BCL-2 and GAPDH as references. The primer pairs (5′-3′) were: IL-1β: Forward ATGCCACCTTTTGACAGTGATG; Reverse TGTGCTGCTGCGAGATTTGA.

TNF-α: Forward CCCTCACACTCACAAACCAC; Reverse ACAAGGTACAACCCATCGGC.

IL-6: Forward AGCCAGAGTCCTTCGAAAGAA; Reverse GCCACTGGTTCTGCTGACTCC.

EGFR: Forward AATGTCTGCCACCTTGCTG; Reverse GCCATTGAACGTACCCGAA.

JUN: Forward GCACATCACCACCTACGAA; Reverse GGGAAGCGTGTTCTGGCTA.

STAT3: Forward ACGAAAGTCGAAAGTTGCTG; Reverse GCTGCCGTTGTTGACTCCT.

PIK3CA: Forward GAACCAGTAGGCAACGTTG; Reverse GCTCTGCTATGAGGCGTTT.

BCL2: Forward GGCCTTCTTTGAGTTCGGTG; Reverse: GGAGAAATCAAACAGGGTCGC.

GAPDH: Forward: GGAGAGTGTTTCCTCGTCCC; Reverse: ATGAAGGGGTCGTTGATGGC. Relative expression was calculated by the 2^-ΔΔCt^ method after reference normalisation.

### Western blotting

Following 24-h treatment, BV2 monolayers in 6-well plates were rinsed twice in ice-cold PBS and lysed on ice in radioimmunoprecipitation assay buffer supplemented with PMSF. The clarified lysates were quantified by bicinchoninic acid, mixed with 5 × loading buffer, and boiled for 10 min. An equal amount of protein (20 μg) was separated using 12% SDS-PAGE (5% stacking gel), transferred to methanol-activated polyvinylidene fluoride, and blocked at 21 °C. The membrane was then incubated 16 h at 4 °C with primary antibodies against EGFR (ab52894, 1:1000), p-EGFR (ab40815, 1:500), c-JUN (ab40766, 1:1000), STAT3 (ab109085, 1:1000), p-STAT3 (ab76315, 1:2000), PIK3CA (ab40776, 1:1000), and BCL-2 (ab182858, 1:1000), all from Abcam (Cambridge, UK). *β*-Actin (ab6276, 1:5000) was used as the loading control. Following Tris-buffered saline with Tween-20 washes, the membranes were incubated for 2 h at 21 °C with HRP-conjugated secondary goat anti-rabbit/mouse IgG-HRP antibodies (bs-0295G-HRP/bs-0296G-HRP, 1:20,000), after which they were developed using enhanced chemiluminescence. Chemiluminescent signals were captured using the JP-K6000 system under non-saturating exposure conditions, and representative exposure times were selected within the linear detection range. Densitometry was performed using ImageJ. Target intensities were normalised first to *β*-actin and then to the LPS model group for inter-blot comparison.

### Apoptosis by Annexin V/PI flow cytometry

Apoptosis was quantified using Annexin V-FITC/propidium iodide dual staining. After treatment, cells were washed with cold PBS and resuspended in Ca^2+^-containing binding buffer. Annexin V-FITC and PI were added per kit instructions; samples were incubated for 15 min at room temperature in the dark and analysed immediately on a flow cytometer (BD FACSCalibur, BD Biosciences; 488 nm excitation). At least 10,000 events were acquired per sample. Data analysis was performed using a sequential gating strategy that included exclusion of debris based on forward scatter/side scatter, singlet discrimination, and quadrant-based analysis of Annexin V-FITC/PI staining to distinguish viable, early apoptotic, late apoptotic, and necrotic populations.

### Fluorescence staining of F-actin and Annexin V-FITC

BV2 cells were seeded on glass coverslips and treated as indicated. For F-actin staining, cells were washed with PBS, fixed with 4% paraformaldehyde for 15 min, permeabilized with 0.1% Triton X-100 for 10 min, and blocked with 5% bovine serum albumin at room temperature for 30 min. Cells were then incubated with Texas Red™-X Phalloidin (Invitrogen, Thermo Fisher) for 30 min at room temperature in the dark. Nuclei were counterstained with DAPI for 5 min. Coverslips were mounted and imaged under identical exposure settings. For Annexin V-FITC fluorescence imaging, treated cells grown on coverslips were washed with PBS and incubated with the Annexin V-FITC Apoptosis Detection Kit working solution in binding buffer for 15 min at room temperature in the dark. Cells were imaged immediately after staining under identical acquisition settings across groups. Representative images were acquired from at least three randomly selected fields for each sample. Quantitative fluorescence-image analysis was performed using ImageJ software (NIH, USA).

### Data statistics

Data are shown as mean ± SD. Normality (Shapiro–Wilk) and homoscedasticity (Levene) were assessed. One-way ANOVA with Tukey’s post-hoc test (or Welch/Games-Howell if unequal variance) was used for ≥3 groups. For families of related endpoints (cytokines), Benjamini-Hochberg FDR control was applied (q < 0.05). A two-sided *p* < 0.05 was considered statistically significant.

## Results

### Network workflow and PSD-anchored target landscape

**Figure 1:**
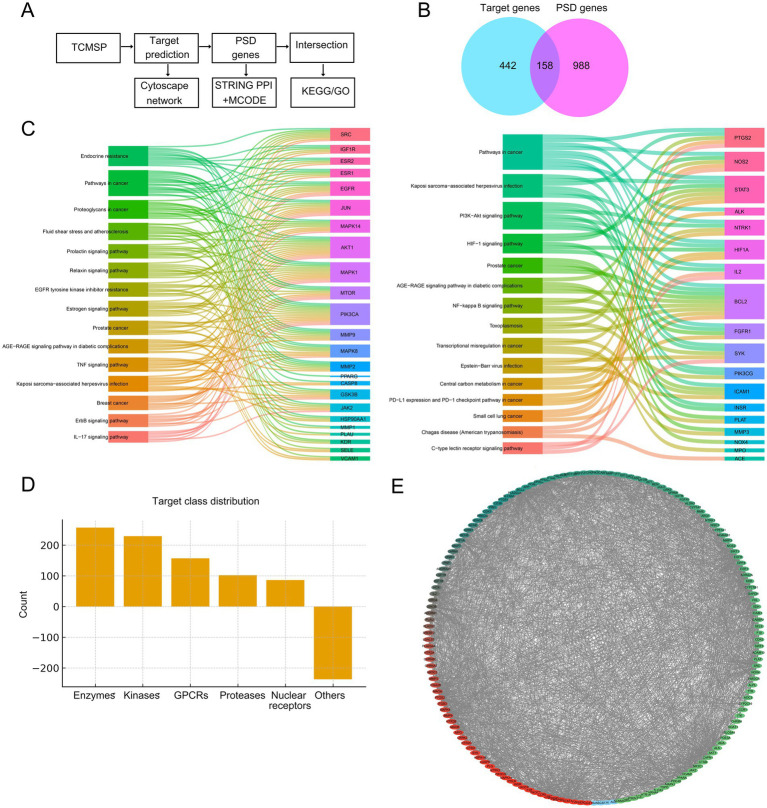
Integrative workflow and PSD-anchored target landscape. **(A)** Analysis pipeline showing constituent curation, target prediction, PSD gene collection, intersection, PPI construction, and KEGG/GO enrichment. **(B)** Venn diagram of predicted targets from the formula and PSD genes, yielding a 158-gene overlap from 442 predicted targets and 988 PSD genes. **(C)** Sankey visualisation linking representative enriched KEGG pathways to their contributing genes, highlighting endocrine and growth-factor signaling, PI3K-AKT, JAK–STAT, NF-κB, HIF-1, adhesion, and cytokine programmes. **(D)** Target class distribution across molecular families, including enzymes, kinases, GPCRs, proteases, nuclear receptors, and others. **(E)** STRING PPI network of the 158 shared genes displayed in a circular layout, edges depict high-confidence interactions, and node clustering indicates hub organisation.

We implemented an integrative workflow (Figure 1A), standardising the constituents of the formula and mapping them to human protein targets. After database integration and deduplication, 35 core active compounds were retained from BADR, yielding 642 nonredundant predicted human targets for subsequent analysis. After normalisation to official gene symbols and removal of unmapped or non-annotated entries, 442 formula-related targets were retained as the final target set for intersection analysis. We then intersected these with PSD-related disease genes and carried the overlap forward for network and enrichment analyses. A total of 1,146 PSD-related genes were collected from the disease database, of which 988 genes remained after deduplication and gene-symbol standardisation. Intersection analysis identified 158 overlapping genes between BADR-predicted targets and PSD-related genes, which were used as the disease-anchored candidate set for downstream analyses. Figure 1B shows a Venn diagram of the 442 putative targets from the formula and the 988 PSD genes, yielding an intersection of 158 genes that grounds subsequent analyses in PSD biology. The pathway-gene Sankey plots (Figure 1C) summarise the top enriched KEGG terms and their contributing genes. These highlight endocrine/ErbB/EGFR-inhibitor resistance, PI3K-AKT, JAK–STAT, NF-κB, HIF-1, and cytokine/adhesion modules that converge on hubs such as EGFR, AKT1, STAT3, JUN, PIK3CA, BCL2, MMPs, ICAM1, and VCAM1. The compound-target network further indicated that several overlapping genes were linked to more than one BADR-derived active compound, supporting the polypharmacological characteristics of the formula. Target-class annotation (Figure 1D) shows the breadth of molecular classes represented, with the most numerous being enzymes and kinases, followed by class-A GPCRs, proteases, and nuclear receptors. The circular network (Figure 1E) visualises the dense connectivity of the 158-gene set, with multiple genes functioning as high-degree hubs, consistent with a polypharmacology topology. Target-class allocation is summarised in Supplementary Table S1, and the full compound-target catalogue underlying the Sankey network is provided in Supplementary Table S2.

### PPI modules and functional programmes prioritised from the 158-gene intersection

**Figure 2:**
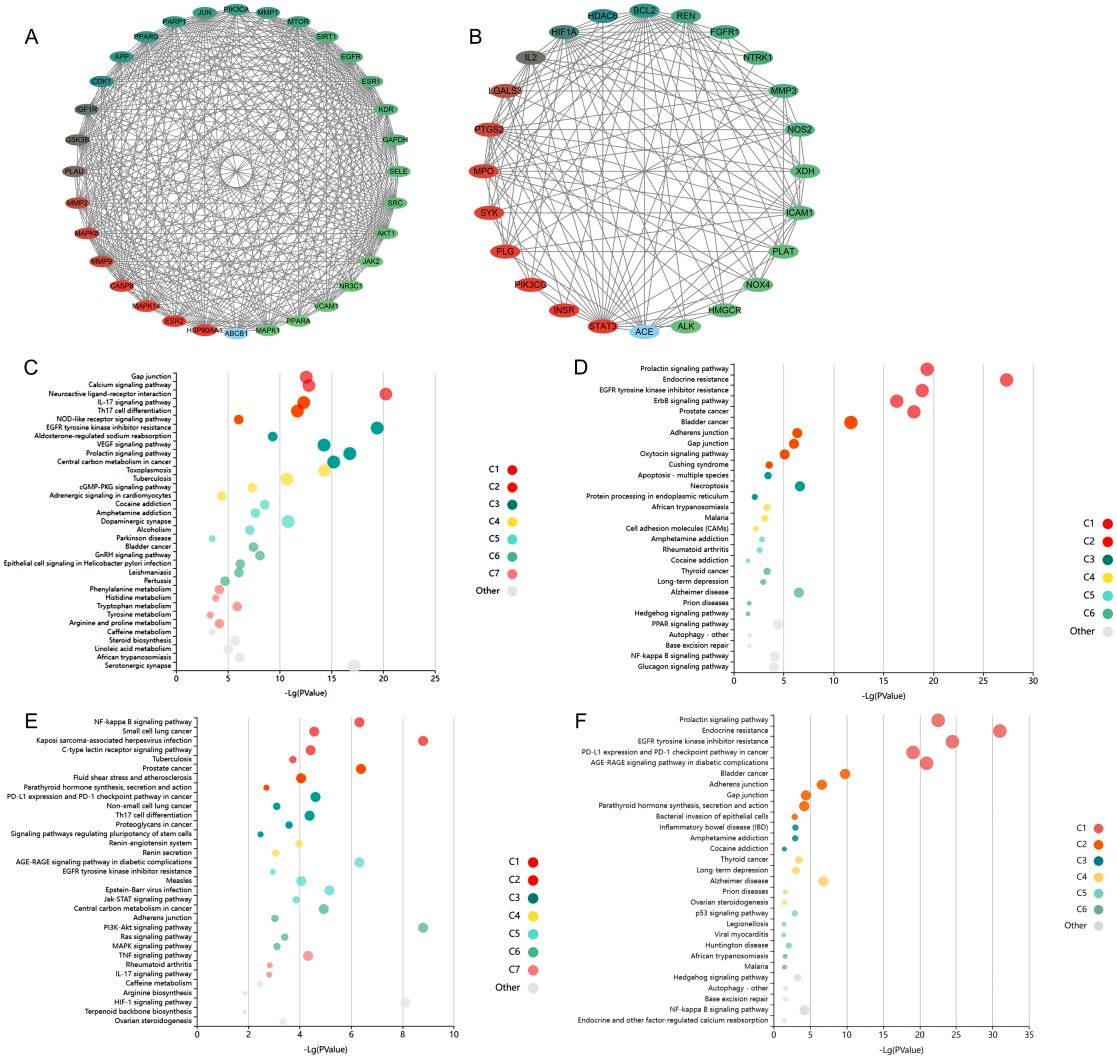
PPI topology, module discovery, pathway enrichment, and hub-target prioritisation. **(A)** MCODE Cluster 1 network enriched for receptor-tyrosine-kinase and stress-chaperone nodes with hubs such as EGFR, AKT1, SRC, JUN, HSP90AA1, ESR1, PPARG, SIRT1, and MMP9. **(B)** MCODE Cluster 2 network centred on inflammatory, survival, and hypoxia nodes including PIK3CA, STAT3, BCL2, HIF1A, with immune-metabolic and neuroactive-ligand components. **(C)** KEGG over-representation for the full 158-gene set. **(D)** KEGG over-representation for Cluster 1 showing dominance of PI3K-AKT, MAPK, adhesion, and ECM remodelling pathways together with receptor tyrosine kinase-linked programmes. **(E)** KEGG over-representation for Cluster 2 highlighting JAK–STAT, NF-κB, HIF-1, cytokine and neuroactive-ligand signaling. **(F)** Functional enrichment analysis of the 28 degree-filtered hub proteins (degree > 20), highlighting the persistence of PI3K-AKT-, JAK–STAT-, NF-κB-, and HIF-1-related signaling among the core candidates.

Projecting the 158-gene set onto STRING yielded a high-confidence PPI map that resolved into two dense MCODE clusters (Figures 2A,B). The two highest-scoring modules were selected for further analysis, with MCODE scores of 24 and 11.652, respectively. NetworkAnalyzer further showed that MCODE 1 contained 32 nodes and 372 edges, whereas MCODE 2 contained 24 nodes and 134 edges, indicating densely connected hub structures within these subnetworks. Cluster 1 contains growth factor and stress hubs (e.g., EGFR, AKT1, SRC, JUN, HSP90AA1, ESR1, PPARG, and SIRT1), suggesting receptor tyrosine kinase inputs and chaperone/stress integration. Cluster 2 contains inflammatory, survival, and hypoxia nodes (PIK3CA, STAT3, BCL2, and HIF1A), as well as immune/metabolic and neuroactive ligand components (ARG1, CCR5, DRD1, HTR1A, HTR1B, HTR2C, SLC6A4, MAOA, and CYP2C19). Based on degree-based topological screening, 28 proteins with degree values greater than 20 were retained as core hub candidates for further prioritisation. KEGG over-representation (Figures 2C–E) reveals the presence of the PI3K-AKT, JAK–STAT, NF-κB, HIF-1, cell adhesion/extracellular matrix, gap junction/calcium signaling, and neuroactive ligand/synaptic pathways across the entire dataset and within the clusters. This indicates coordinated crosstalk rather than isolated hits. Further enrichment analysis of the 28 degree-filtered hub proteins (Figure 2F) confirmed that these core candidates remained strongly associated with inflammatory-survival signaling pathways. Among the highly connected genes, EGFR, STAT3, BCL2, SYK, SRC, JUN, MAPK1, HIF1A, PTGS2, and PIK3CA represented the top 10 most connected regulatory nodes, while EGFR, STAT3, PTGS2, SYK, and PIK3CA showed the strongest compound-association profiles in the compound-target network. Notably, EGFR, STAT3, PIK3CA, JUN, and BCL2 were all included in the core hub set and were therefore prioritised for downstream validation on the basis of topological importance, pathway relevance, and direct association with BADR-derived active compounds, as summarised in Supplementary Table S3. Together, the module structure and enrichment patterns identify an axis linking upstream receptor tyrosine kinases to transcriptional effectors and survival checkpoints, which we investigate in subsequent experiments.

### LC–MS/MS-based chemical characterisation of BADR extract

**Figure 3:**
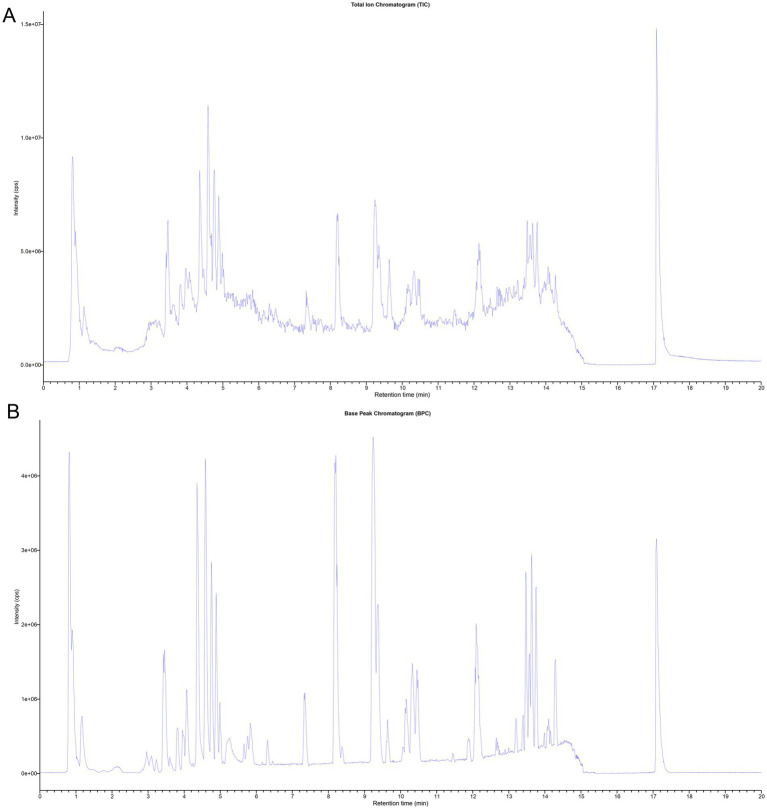
LC–MS/MS-based chemical characterisation of BADR extract. **(A)** Representative total ion chromatograms of three independently prepared BADR samples. **(B)** Representative base peak chromatograms of three independently prepared BADR samples.

To characterise the actual BADR extract used in the biological experiments, LC–MS/MS analysis was performed on three independently prepared BADR samples. Representative total ion chromatograms showed highly similar overall peak patterns across the three samples (Figure 3A). Consistently, the base peak chromatograms also displayed comparable chromatographic profiles among the independently prepared samples (Figure 3B). Batch-level fingerprint consistency analysis further showed concordant profiles across the three BADR samples. LC–MS/MS-based constituent annotation identified multiple putative compounds in the BADR extract, and the detailed annotated constituent list is provided in Supplementary Table S4. Together, these results provide chemical characterisation of the experimental BADR extract and support its batch-level consistency for the subsequent biological assays.

### LPS induces dose-dependent microglial activation with cytokine escalation and morphological remodelling

**Figure 4:**
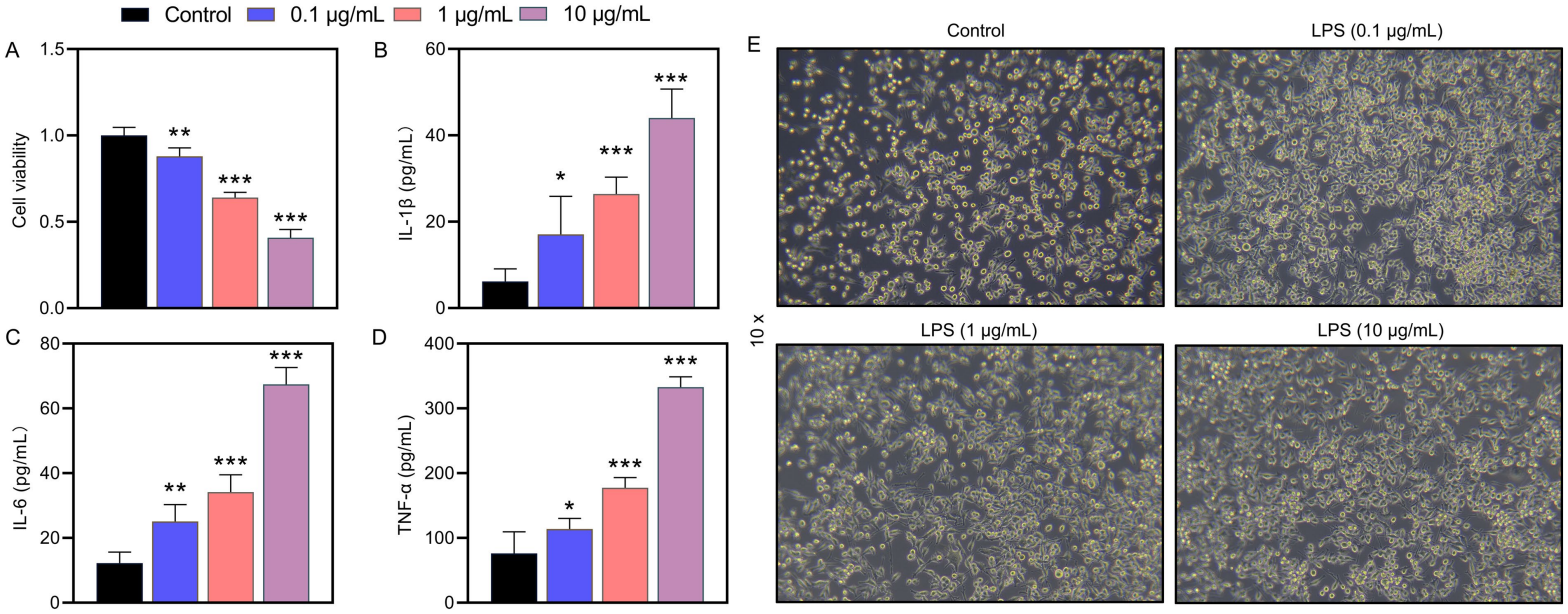
LPS challenge in BV2 cellular function. **(A)** CCK-8 assay measuring cell viability of BV2 cells exposed to LPS at 0.1, 1, and 10 μg/mL for 24 h. **(B–D)** ELISA quantifying IL-1*β*, IL-6, and TNF-*α* in culture supernatants from the same treatments. **(E)** Phase-contrast micrographs (10 × objective) documenting cellular morphology in Control and LPS-treated groups at the indicated doses. *p < 0.05, **p < 0.01, ***p < 0.001.

Exposure to increasing concentrations of LPS (0.1–10 μg/mL for 24 h) resulted in a graded decrease in BV2 viability, providing a controllable inflammatory/toxic burden for downstream assays (Figure 4A). Secreted IL-1β, IL-6, and TNF-*α* increased in parallel, with significant markers in the plots denoting stepwise increases relative to the untreated control at each dose (Figures 4B–D). Phase-contrast imaging captured the expected activation-like morphology, including process retraction, soma rounding, and increased cell compactness, which intensified as the LPS dose increased (Figure 4E). Together, these data verify a robust model of microglial activation with concordant molecular and phenotypic readouts, providing a quantitative context for testing pharmacological modulation.

### BADR defines a non-cytotoxic window and improves viability relative to the LPS model

**Figure 5:**
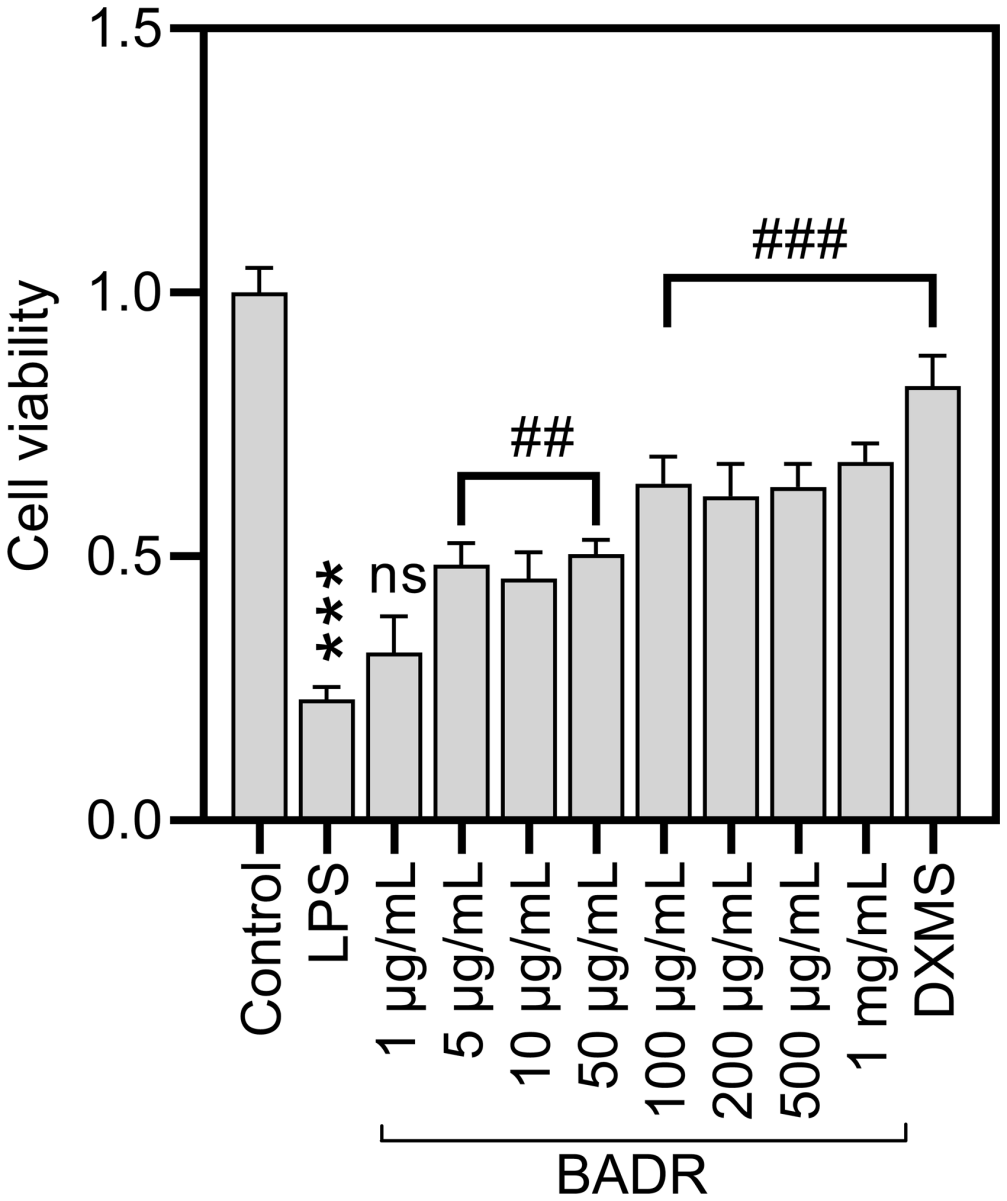
BADR dose–response on BV2 viability under inflammatory stress. CCK-8 assay measuring cell viability in Control, LPS model, LPS plus graded BADR concentrations (1 μg/mL to 1 mg/mL), and LPS plus the reference drug DXMS. ***p < 0.001 vs. Control; ##*p* < 0.01, ###*p* < 0.001 vs. LPS Model; ns, not significant vs. LPS.

A dose–response screen of the BADR formula showed preserved viability over a broad range in BV2 cultures, as well as progressive recovery of cell viability when applied to LPS-challenged cells (Figure 5). The curve demonstrates monotonic improvement across the tested concentrations, while the positive control DXMS produced the anticipated rescue effect within the same experimental framework. Significance annotations indicate differences versus LPS and trends across the BADR series. These results establish a working, non-cytotoxic dosing window and justify the concentrations selected for confirmatory anti-inflammatory and anti-apoptotic assays.

### BADR reduces apoptosis and dampens the LPS secretome with benchmark-level directionality

**Figure 6:**
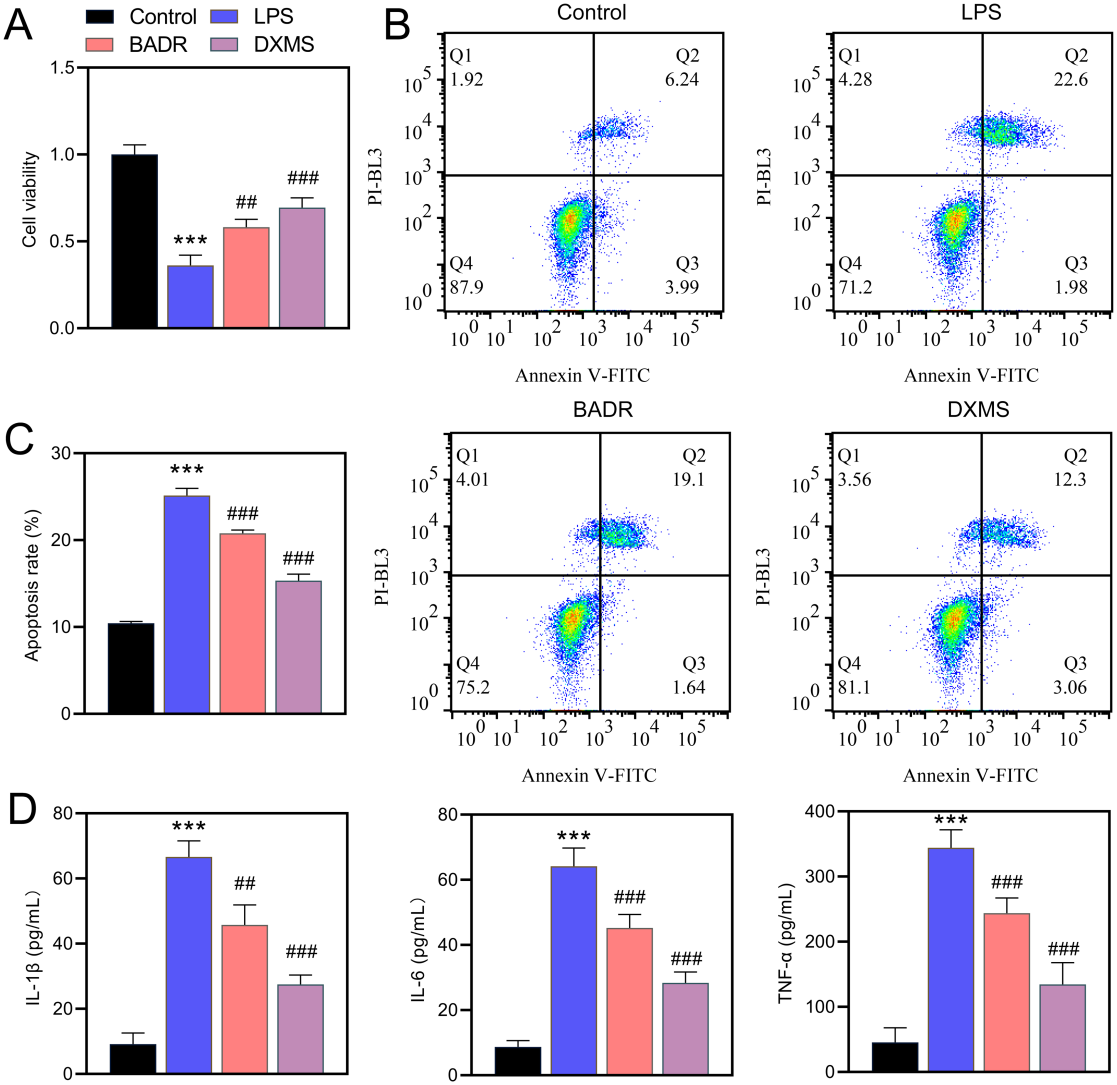
Viability, apoptosis, and cytokines in LPS-challenged BV2 cells with BADR or DXMS. **(A)** CCK-8 assay measuring cell viability in four groups: Control, LPS, LPS + BADR, and LPS + DXMS. **(B,C)** Annexin V-FITC/PI flow cytometry for the same groups, with a shared gating strategy (debris exclusion, singlets, four quadrants). **(D)** ELISA quantifying IL-1β, IL-6, and TNF-α in matched supernatants. ***p < 0.001 vs. Control; ##*p* < 0.01, ###*p* < 0.001 vs. LPS Model.

CCK8 results shown that BADR increased BV2 viability relative to the LPS model (Figure 6A) and reduced the total apoptotic fraction using Annexin V/PI flow cytometry (Figures 6B,C). Dot-plot quadrant distributions showed reduced early and late apoptotic populations compared with LPS alone. In the same samples, ELISA demonstrated a consistent decrease in IL-1β, IL-6, and TNF-*α* following BADR treatment (Figure 6D). The direction and magnitude of this decrease were similar to those achieved by DXMS, indicating that BADR is effective in the treatment of microglial inflammation. The alignment of viability, apoptosis, and cytokine outputs indicates that BADR acts upstream of shared pro-inflammatory nodes rather than on isolated terminal effectors.

### BADR preserves cytoskeletal architecture and limits apoptotic staining in activated BV2 cells

**Figure 7:**
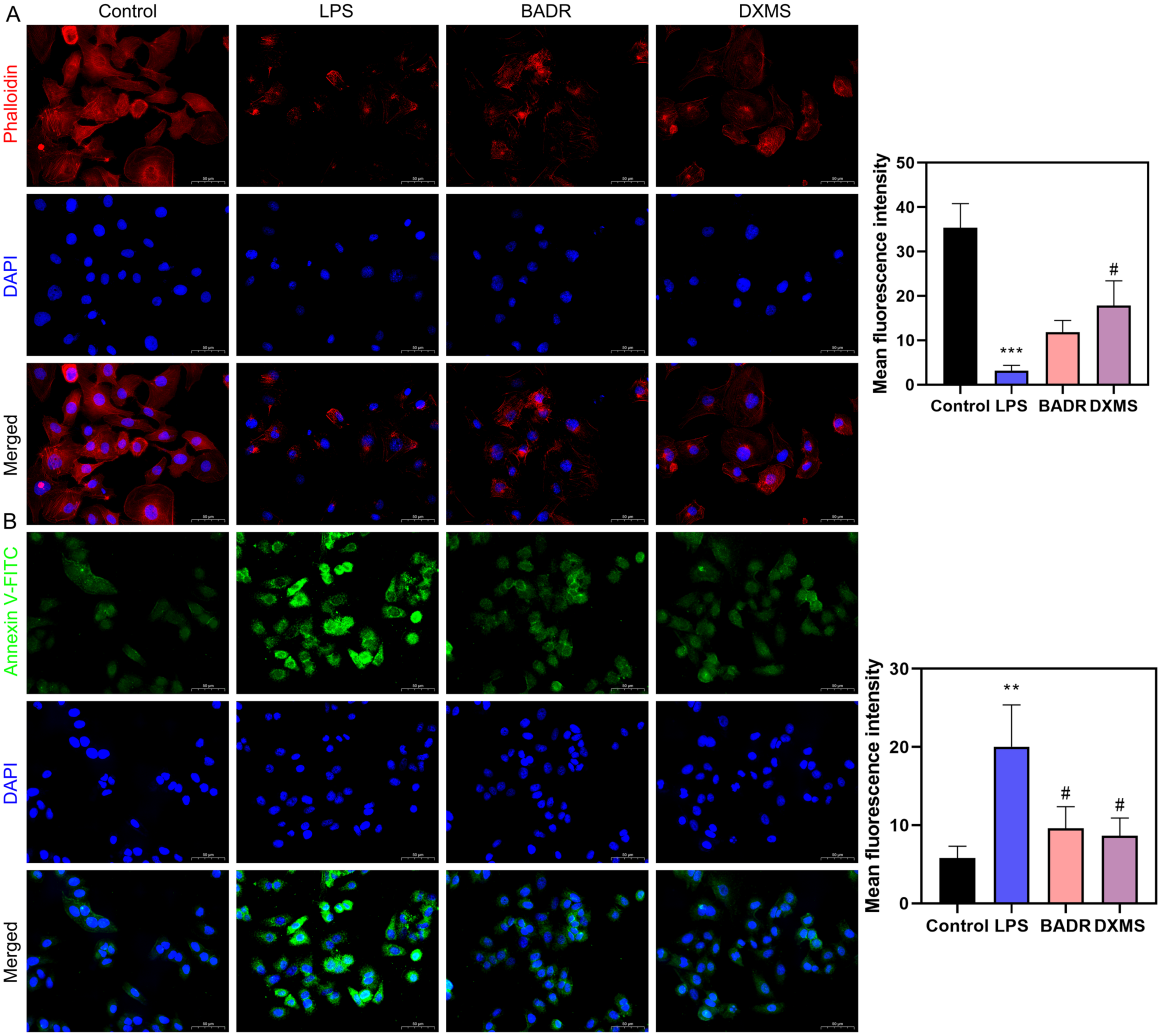
Fluorescence imaging of cytoskeleton and apoptosis in BV2 cells. **(A)** Phalloidin (F-actin, red), DAPI (nuclei, blue), and merged images for Control, LPS, LPS + BADR, and LPS + DXMS groups. **(B)** Annexin V-FITC (green), DAPI (blue), and merged images in the same groups to visualise apoptotic membrane phosphatidylserine exposure. Scale bars = 50 μm. **p < 0.01, ***p < 0.001 vs. Control; #*p* < 0.05 vs. LPS model.

Fluorescence imaging with phalloidin (F-actin) and DAPI revealed intact stress fibre organisation and normal nuclear morphology in control cells. In contrast, LPS produced fragmented and attenuated actin structures, as well as nuclear condensation. BADR restored orderly F-actin bundles and improved cell spreading, approaching the morphology observed with DXMS (Figure 7A). Annexin V-FITC staining revealed extensive phosphatidylserine exposure on the cell surface after LPS treatment, which is consistent with the initiation of apoptosis; this signal was visibly reduced in the BADR group and further diminished in the DXMS group (Figure 7B). The qualitative concordance between cytoskeletal stabilisation and reduced annexin signal supports the protective effect of BADR on the structure–function coupling of microglia under inflammatory stress.

### BADR recalibrates the EGFR-PIK3CA-JUN-STAT3-BCL2 axis at transcript/protein levels

**Figure 8:**
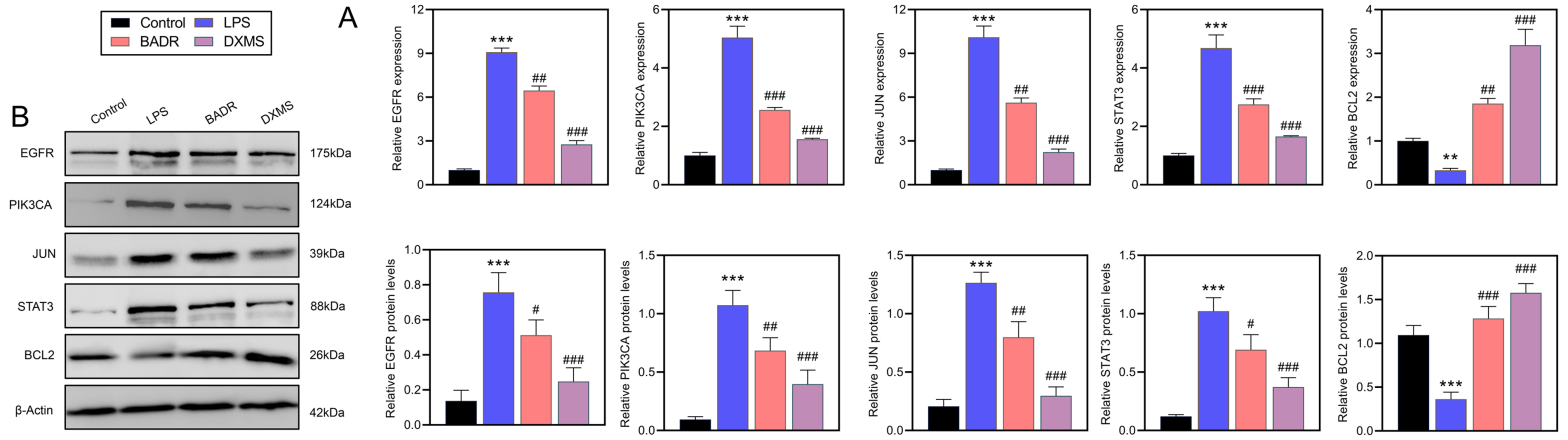
Transcriptional and protein profiling of EGFR-PIK3CA-JUN-STAT3-BCL2. **(A)** qRT-PCR analysis measuring mRNA expression of BCL-2, EGFR, PIK3CA, JUN, and STAT3 in Control, LPS, LPS + BADR, and LPS + DXMS groups. **(B)** Western blotting of the corresponding proteins (BCL-2, EGFR, PIK3CA, JUN, STAT3) with β-actin as loading control. **p < 0.01, ***p < 0.001 vs. Control; #*p* < 0.05, ##*p* < 0.01, ###*p* < 0.001 vs. LPS Model.

The qRT-PCR demonstrated that LPS increased the transcripts of EGFR, PIK3CA, JUN, and STAT3, while decreasing the relative level of BCL-2. BADR shifted all five genes toward homeostatic levels, lowering EGFR/PIK3CA/JUN/STAT3 and restoring BCL-2. Statistical markers in the plots indicated significant differences versus LPS, and DXMS mirrored these trends (Figure 8A). Western blots reproduced the directionality observed at the transcript level at the protein level: the higher bands for EGFR, PIK3CA, JUN, and STAT3 observed in the LPS model were attenuated by BADR, whereas BCL-2 increased relative to LPS (Figure 8B). Multi-layer analysis links cytokine control to a mechanistic axis centred on receptor-kinase input and transcriptional effectors with downstream survival checkpoints.

### EGF partially reverses BADR-mediated suppression of EGFR/STAT3 activation and inflammatory cytokine release

**Figure 9:**
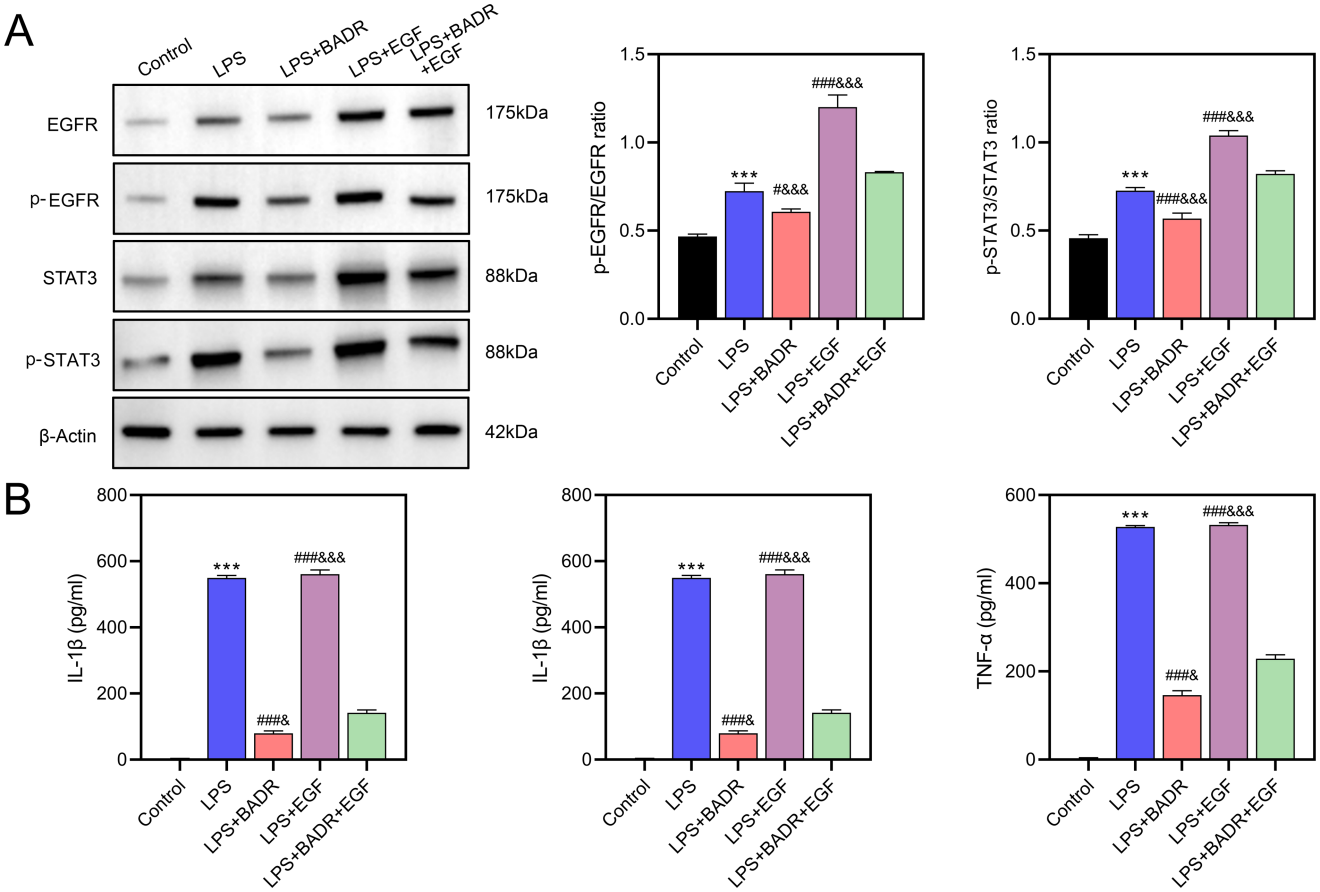
EGF rescue experiment in LPS-stimulated BV2 cells. **(A)** Representative Western blot bands of EGFR, p-EGFR, STAT3, and p-STAT3 in the Control, LPS, LPS + BADR, LPS + EGF, and LPS + BADR+EGF groups. **(B)** ELISA analysis of IL-1β, TNF-α, and IL-6 in cell culture supernatants from the indicated groups. ***p < 0.001 vs. Control; ###*p* < 0.001 vs. LPS Model; &*p* < 0.01, &&&*p* < 0.001 vs. LPS + BADR+EGF.

To further assess whether the anti-inflammatory effect of BADR was associated with the EGFR/STAT3 axis, an EGF-based rescue experiment was performed in LPS-stimulated BV2 cells. Compared with the Control group, LPS stimulation increased EGFR and STAT3 expression and markedly enhanced EGFR and STAT3 phosphorylation. BADR treatment attenuated these changes, whereas EGF treatment alone further increased EGFR and STAT3 activation under LPS stimulation. When EGF was added to BADR-treated cells, EGFR and STAT3 expression and phosphorylation were partially restored compared with the LPS + BADR group, indicating that reactivation of EGFR signaling could weaken the inhibitory effect of BADR on this pathway (Figure 9A). Consistent with these signaling changes, ELISA showed that BADR significantly reduced the LPS-induced release of IL-1β, TNF-*α*, and IL-6, whereas EGF increased the levels of these cytokines. In the LPS + BADR+EGF group, IL-1β, TNF-α, and IL-6 levels were significantly higher than those in the LPS + BADR group, but remained lower than those in the LPS + EGF group (Figure 9B). These findings indicate that EGF partially reverses the suppressive effects of BADR on EGFR/STAT3 activation and downstream inflammatory cytokine release, supporting the involvement of the EGFR/STAT3 axis in the BADR response.

## Discussion

Considerable evidence suggests that PSD is increasingly recognised as a neuroimmune complication of cerebrovascular injury, whereby microglial activation and cytokine dysregulation hinder recovery and destabilise mood circuitry (5, 27). Our findings converge on a coherent mechanism in which a multi-component BADR formula attenuates inflammatory signaling in microglia while preserving programmes that promote cell survival through nodes that were independently prioritised by our network analysis. At the computational level, intersecting TCMSP-mapped constituents with a PSD gene universe yielded a disease-anchored set enriched for receptor tyrosine kinases and downstream kinase-transcription factor relays. Here, EGFR, PIK3CA/AKT, and STAT3/JUN emerged as plausible control points, which are also emphasised in recent neuroinflammation literature. At the experimental level, the same axis was reflected across secreted outputs, molecular markers, and cell fate readouts in LPS-activated microglia, aligning with contemporary views of JAK/STAT and PI3K/AKT as shared conduits from receptor input to cytokine transcription and apoptosis control (17, 29). Internal agreement among cytokines, transcripts, total proteins, and apoptosis indicates that the intervention rebalances both upstream receptor-kinase pressure and downstream survival checkpoints, rather than acting on a single terminal effector. Nevertheless, unlike animal models of post-stroke depression, which can address behavioural alterations, brain-region specificity, neuronal injury, synaptic remodelling, and neurogenesis-related changes after stroke, the BV2/LPS system does not recapitulate the full pathological spectrum of PSD. Its value lies in its simplicity, stability, and suitability for early-stage mechanistic screening of microglia-associated inflammatory signaling under controlled conditions. Therefore, our findings should be interpreted as mechanism-oriented evidence obtained in a PSD-relevant microglial inflammatory context, rather than as direct proof of therapeutic efficacy across the full pathological spectrum of PSD.

The LPS qualification series provides the basis for interpretation. Increasing exposure resulted in the anticipated decrease in BV2 viability, accompanied by graded increases in IL-1β, IL-6, and TNF-*α*, as well as the typical morphological changes of process retraction and soma rounding associated with activation. These concordant cellular and secretory responses confirm that the model captures a high inflammatory load in microglia, supports quantitative comparisons across treatment arms, and defines a dynamic range wide enough to detect pharmacological rescue (30, 31). The formula’s ability to temper this secretory phenotype is consistent with current reports showing that small molecules and natural products can suppress LPS-induced cytokines in microglia, as well as with the anti-inflammatory properties exemplified by steroid benchmarks (32). In this context, the BADR dose–response curve demonstrates that the formulation is well tolerated across the working range, promoting survival in LPS-challenged cultures. This establishes a safe and effective window for mechanistic studies, avoiding the interpretative issue of extract-induced cytotoxicity.

The inclusion of DXMS as a positive comparator helped evaluate whether BADR modified shared inflammatory drivers rather than isolated terminal effectors. In the presence of LPS, BADR increased CCK-8 viability and reduced total apoptotic fractions as determined by Annexin V/PI flow cytometry. The dot-plot distributions indicate coordinated decreases in both the early and late apoptotic compartments. This argues against a trivial shift between phases, instead supporting a genuine survival benefit. In the same samples, BADR lowered all three quantified cytokines, and the directionality broadly paralleled that of the glucocorticoid benchmark. It is difficult to achieve convergence of viability, apoptosis, and secreted cytokines by targeting a single terminal mediator; this pattern is more consistent with the attenuation of upstream inputs that simultaneously regulate survival thresholds and inflammatory transcription (33–35). Fluorescence imaging provides further support for this interpretation at the structural level. LPS disrupted F-actin architecture and produced nuclear condensation consistent with stress and apoptotic priming, whereas BADR preserved stress-fibre organisation and improved cell spreading. The reduction in Annexin V-FITC staining in the same imaging series is consistent with the flow cytometry data, linking cytoskeletal stabilisation to decreased phosphatidylserine externalisation. As actin dynamics influence receptor trafficking and signalosome assembly in microglia, the morphological rescue provides a plausible physical context for the changes observed in upstream signaling nodes (36).

The observed transcriptional and protein-level rebalancing of EGFR, PIK3CA/AKT, STAT3/JUN, and BCL2 is mechanistically plausible because these nodes occupy well-defined positions at the interface of receptor-mediated neuroinflammatory signaling and cell-survival regulation. EGFR sits at the interface of growth-factor sensing and metabolic resilience, relaying signals to PIK3CA/AKT. Its broader central nervous system relevance and links to injury responses have recently been reviewed, reinforcing its plausibility as an upstream gate in glia (37). STAT3 mediates cytokine-receptor inputs and, together with JUN, integrates stress stimuli to reprogram gene expression. This places JAK–STAT and AP-1 programmes at the heart of microglial inflammatory control (17). BCL2 represents a nodal checkpoint for survival that is consistent with PI3K/AKT-conditioned apoptotic thresholds and aligns with current syntheses of BCL2 family biology (38). Taken together, these targets support the idea that an intervention with balanced activity across the EGFR-JAK/STAT-PI3K/AKT pathways and related checkpoints could reduce inflammatory amplification, preserve survival programmes, and stabilise neuro-modulatory circuits that are affected in PSD. Here, we observed that LPS upregulates EGFR, PIK3CA, JUN, and STAT3, while suppressing BCL2. BADR reverses each of these effects at both the mRNA and protein levels. The coherence across layers suggests that assay noise is not a factor and indicates true pathway rebalancing. One possible model is that BADR reduces receptor-kinase pressure at EGFR, which has an effect on PI3K catalytic subunits. This lowers downstream stress-responsive transcription via STAT3 and JUN, and relieves the anti-apoptotic checkpoint BCL-2 from suppression (39). The restoration of BCL-2 occurs at the same time as decreased annexin positivity and increased viability. This suggests that the anti-inflammatory effect of BADR is coupled to the preservation of mitochondrial integrity rather than being achieved at the expense of cell survival. Immunofluorescence colocalisation provides an additional qualitative layer. In LPS-treated cells exposed to BADR, an intracellular green signal appeared in microdomains that overlapped with EGFR and STAT3. The absence of this signal in untreated or non-BADR conditions makes non-specific background less likely, and the spatial overlap is compatible with the idea that the formulation’s constituents are present within compartments where receptor and transcriptional signaling converge. While these images do not prove direct binding or obligatory physical interaction, they are consistent with a scenario in which BADR gains intracellular access under inflammatory conditions and influences the abundance, distribution, or turnover of EGFR- and STAT3-containing complexes. The most parsimonious mechanistic interpretation is that the BADR formula reduces receptor-kinase activity centred on EGFR, with feed-through to PI3K/AKT, while also moderating STAT3/JUN-linked transcriptional programmes. This results in lower cytokine output and preservation of BCL2-associated survival, which is consistent with a previous study (39). Contemporary syntheses of JAK/STAT in brain inflammation and of BCL2-family control over mitochondrial apoptosis provide independent support for this sequence, and focused reviews on microglial PI3K/AKT further reinforce the placement of survival gating downstream of receptor and cytokine cues.

This study has limitations. First, the cellular system relies on an LPS-activated BV2 model and therefore addresses only a PSD-relevant inflammatory component rather than the full pathological spectrum of post-stroke depression. In particular, no *in vivo* PSD model was included, making it impossible to determine whether BADR exerts therapeutic effects on behavioural phenotypes or brain-level pathology in animals. Second, the study is limited in its ability to resolve mechanisms, focusing solely on transcripts and total proteins. We did not quantify the phosphorylation states of EGFR/JAK/STAT3/AKT, the nuclear translocation of STAT3, or the feedback of SOCS3. Furthermore, time-course analyses were not performed to contextualise the intervention within pathway kinetics. Third, although the EGF rescue experiment provides preliminary evidence supporting the involvement of the EGFR/STAT3 axis, the causal dependence on this pathway has not been fully established. Additional pharmacological inhibition or genetic perturbation experiments, such as EGFR or JAK/STAT blockade, IL-6 rescue, siRNA knockdown, or overexpression approaches, are still needed to more definitively link signaling normalisation to cytokine and apoptosis outcomes. Fourth, the current target prioritisation is based on network topology, pathway relevance, compound-target connectivity, and cellular validation, but direct biochemical target-engagement evidence remains lacking. Orthogonal approaches, such as CETSA/thermal shift assays, limited proteolysis, SPR/MST, pharmacological blockade, or genetic perturbation, will be needed to further validate target engagement. Although LC–MS/MS provided preliminary chemical characterisation and supported batch-level consistency of the BADR extract, fully quantitative chemical standardisation and more comprehensive quality-control assessments, including endotoxin testing and systematic pH/osmolality verification, remain needed for translational rigour. Finally, we did not evaluate *in vivo* efficacy, brain exposure, behavioural outcomes, or monoaminergic/synaptic readouts in PSD-relevant animal models. This leaves the link between modulation of microglia and network-level and behavioural benefits untested. These are the directions and goals of our future work.

## Conclusion

The present study shows that BADR attenuates LPS-induced inflammatory injury in BV2 microglia by reducing IL-1β, IL-6, and TNF-*α* levels, improving cell viability, decreasing apoptosis, and maintaining cytoskeletal integrity. BADR also rebalanced the EGFR-PIK3CA-JUN-STAT3-BCL2 axis at the transcript and protein levels, while spatial evidence suggested that the formulation localised to signaling-rich microdomains. These findings provide mechanism-oriented evidence that BADR may modulate microglia-associated inflammatory and survival signaling in a PSD-relevant context. Future studies should further verify phosphorylation dynamics and target engagement and, importantly, evaluate BADR in ischemia-related in vivo PSD models with behavioural, brain-exposure, neuronal, synaptic, and neurogenesis-related outcomes.

## Data Availability

The raw data supporting the conclusions of this article will be made available by the authors, without undue reservation.

## Supplementary material

SUPPLEMENTARY TABLE S1 Distribution of predicted human targets by class for the formula.

SUPPLEMENTARY TABLE S2 Complete compound-target mapping underlying the Sankey network, listing compound name, target gene symbol, and database identifiers.

SUPPLEMENTARY TABLE S3 Summarises the prioritisation of EGFR, STAT3, PIK3CA, JUN, and BCL2 based on overlap analysis, degree-filtered hub screening, and compound-target connectivity.

SUPPLEMENTARY TABLE S4 Annotated LC-MS/MS-identified constituents of BADR extract.
