# Supplementary material for: Blood-activating, depression-relieving formula alleviates post-stroke depression: mechanistic insights from network pharmacology and microglial validation
Source: Front Neurol. 2026 Jul 2;17:1780535. doi: 10.3389/fneur.2026.1780535 (PMC13372893; doi:10.3389/fneur.2026.1780535)

Figure 8B
EGFR
175 kDa

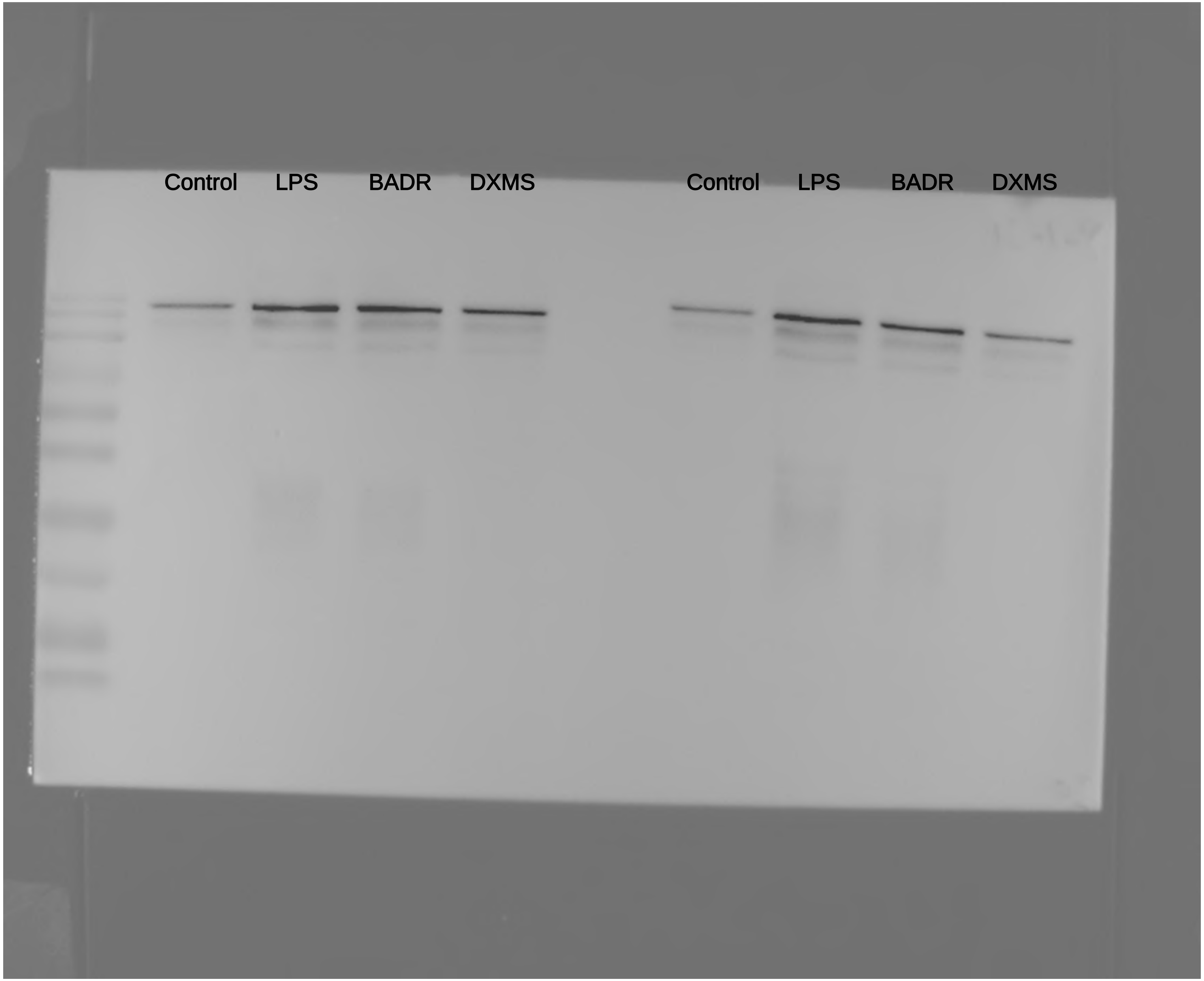

Figure 8B
EGFR
175 kDa

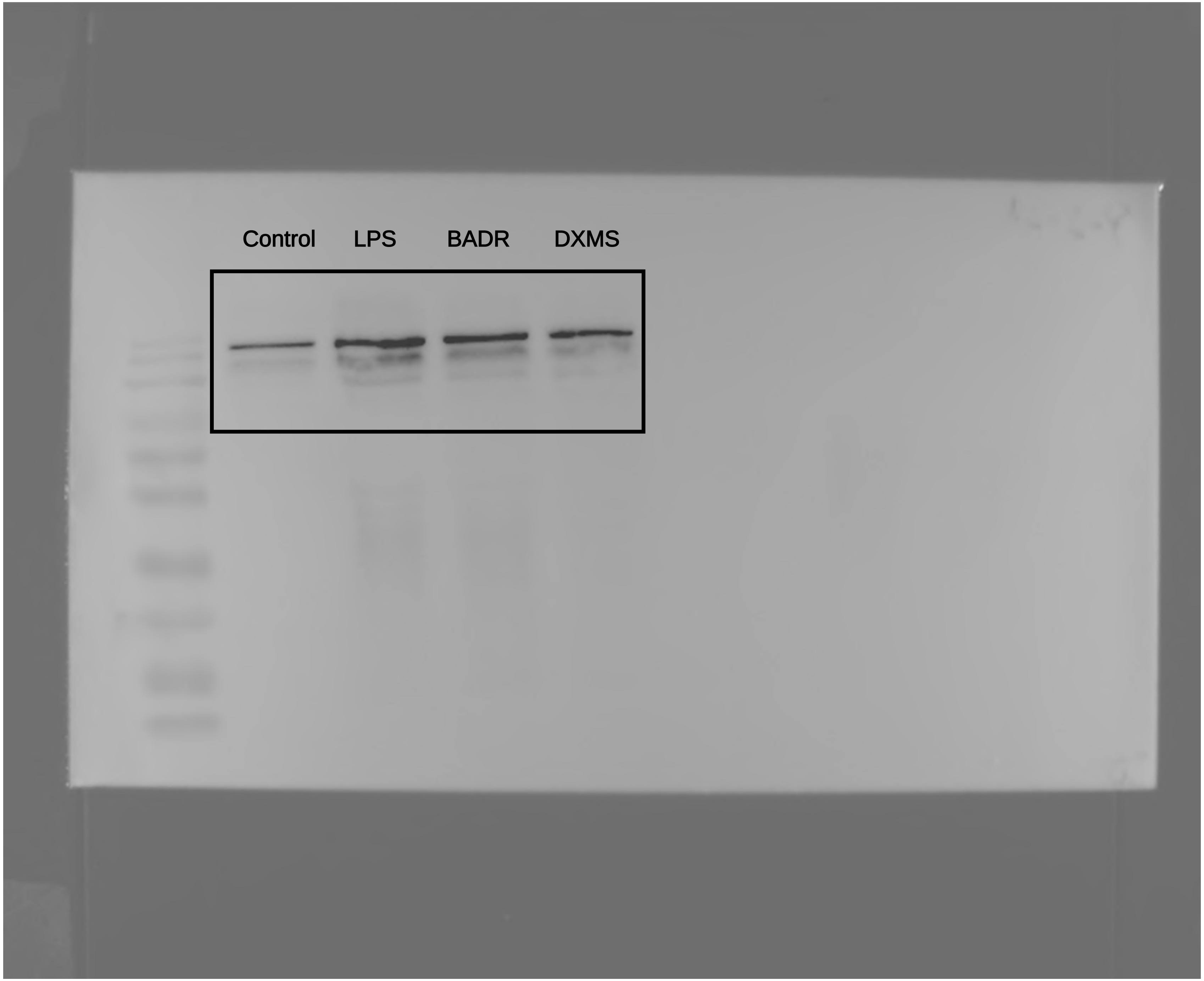

Figure 8B
PIK3CA
124 kDa

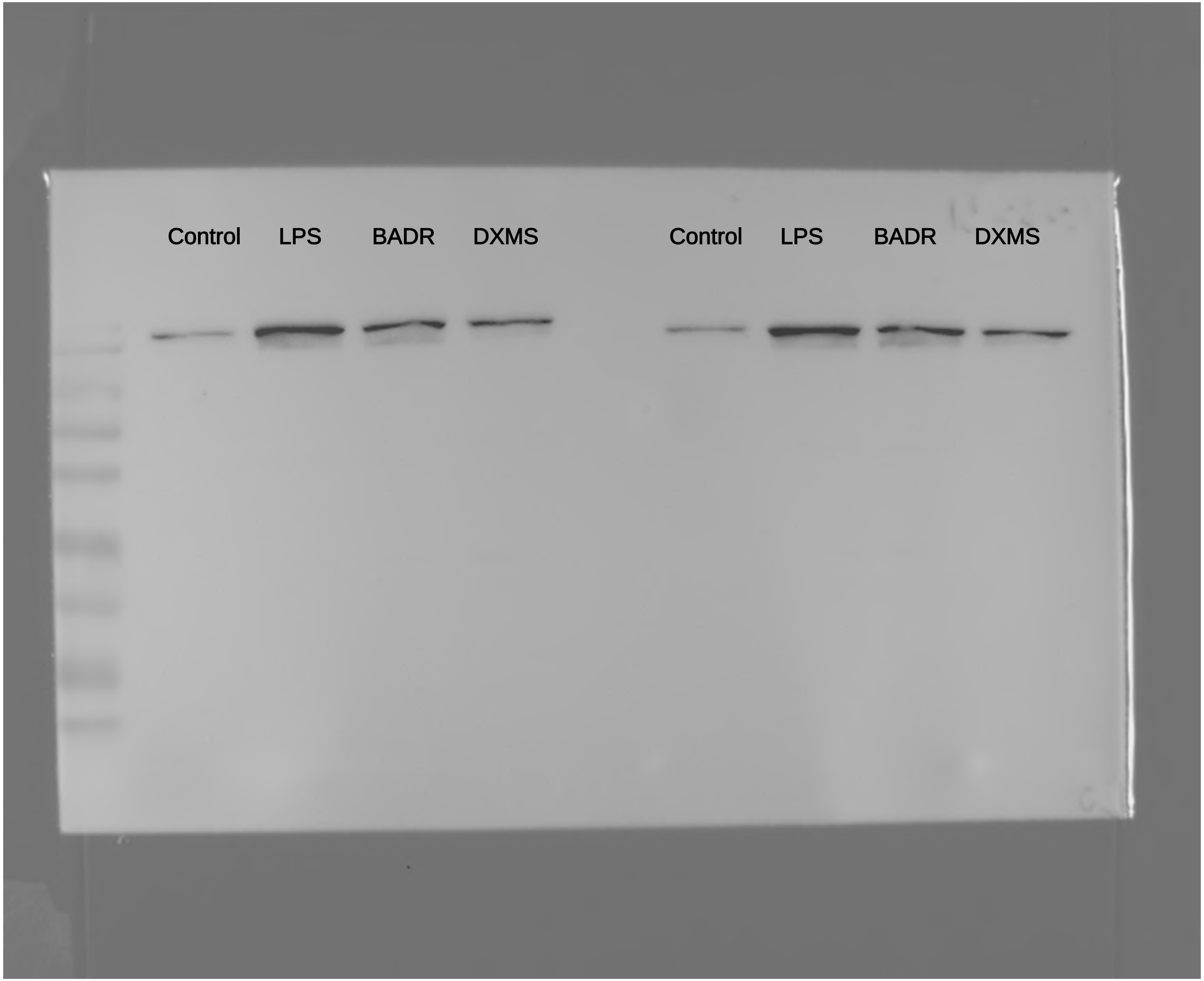

Figure 8B
PIK3CA
124 kDa

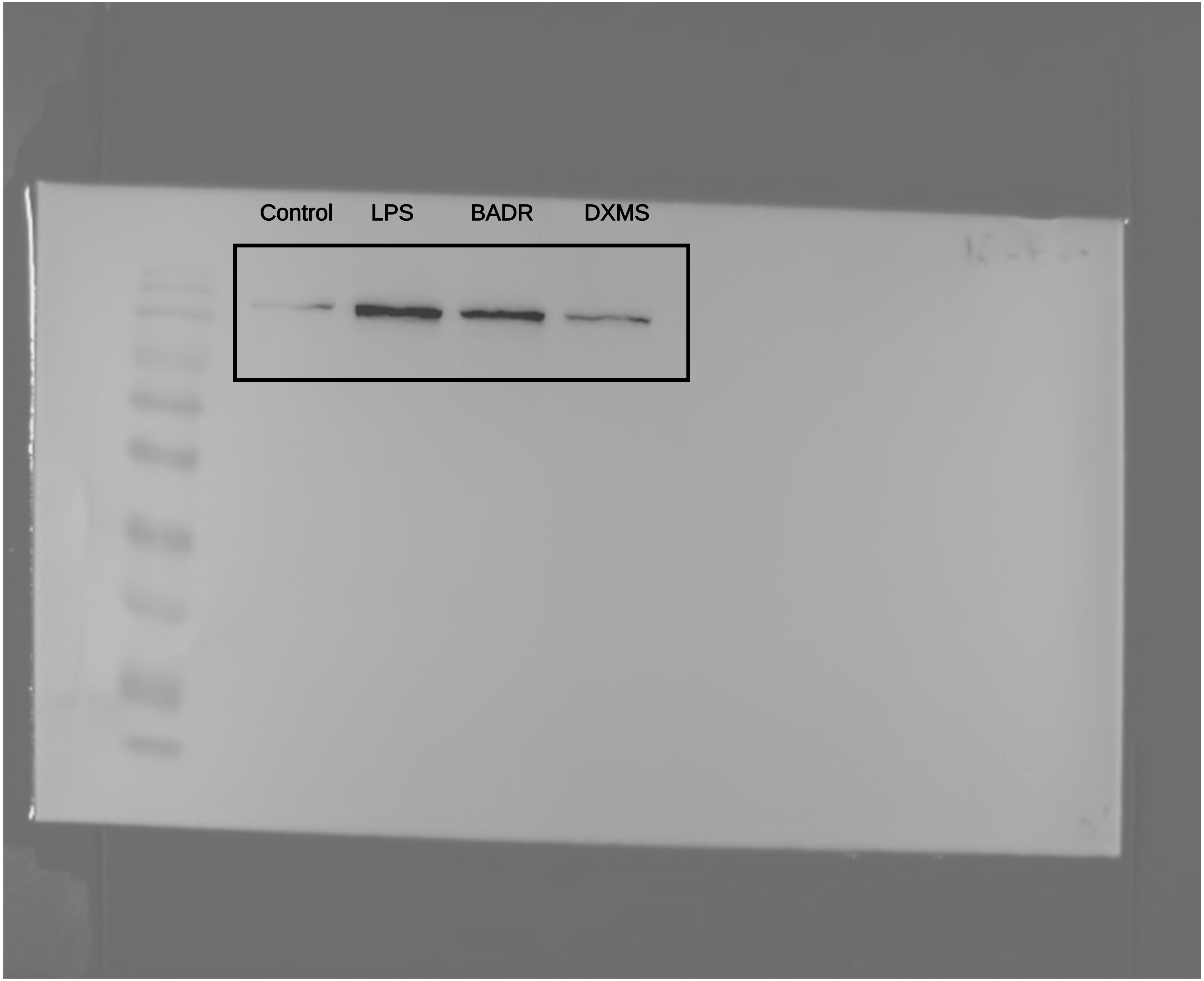

Figure 8B
JUN
39 kDa

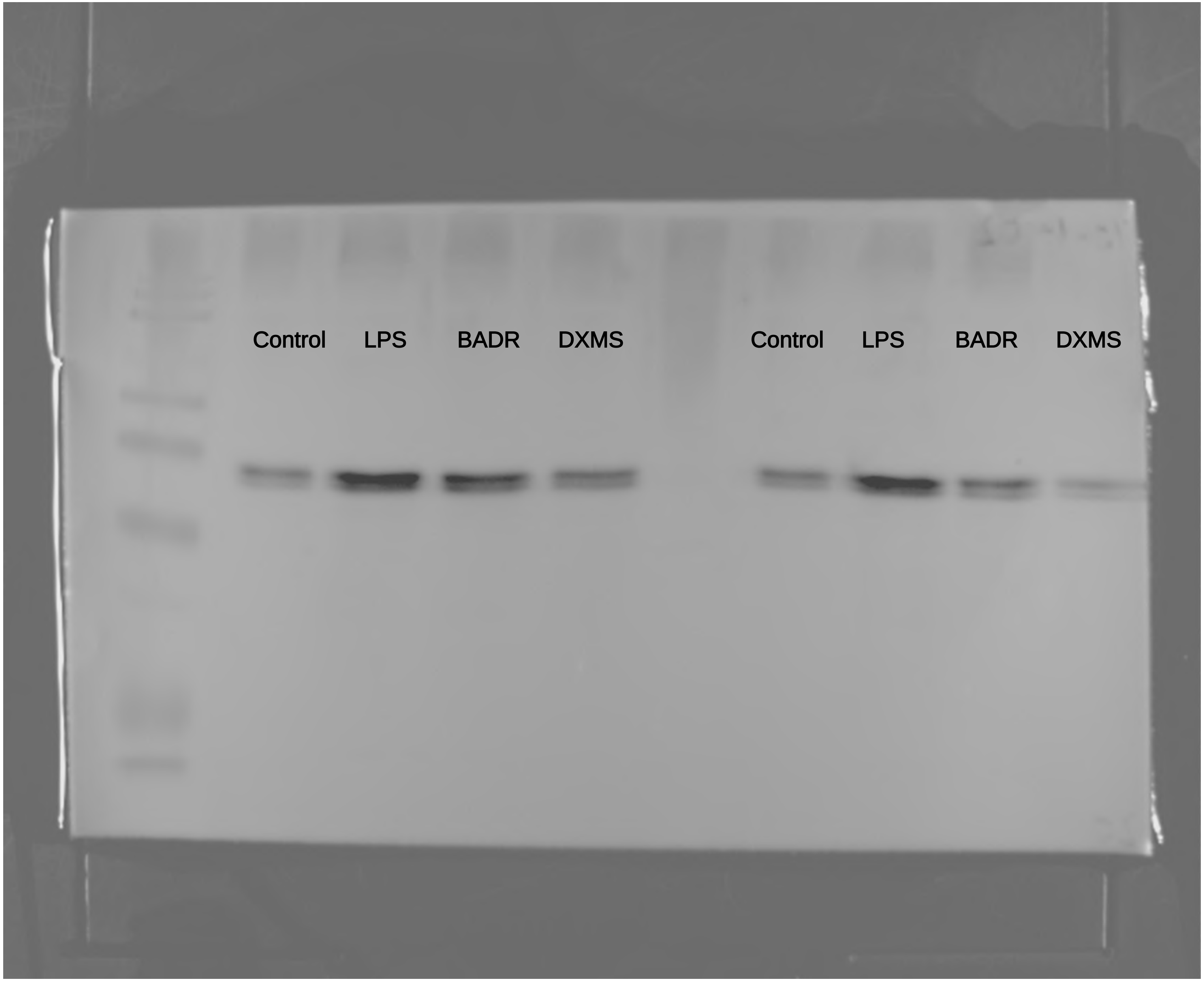

Figure 8B
JUN
39 kDa

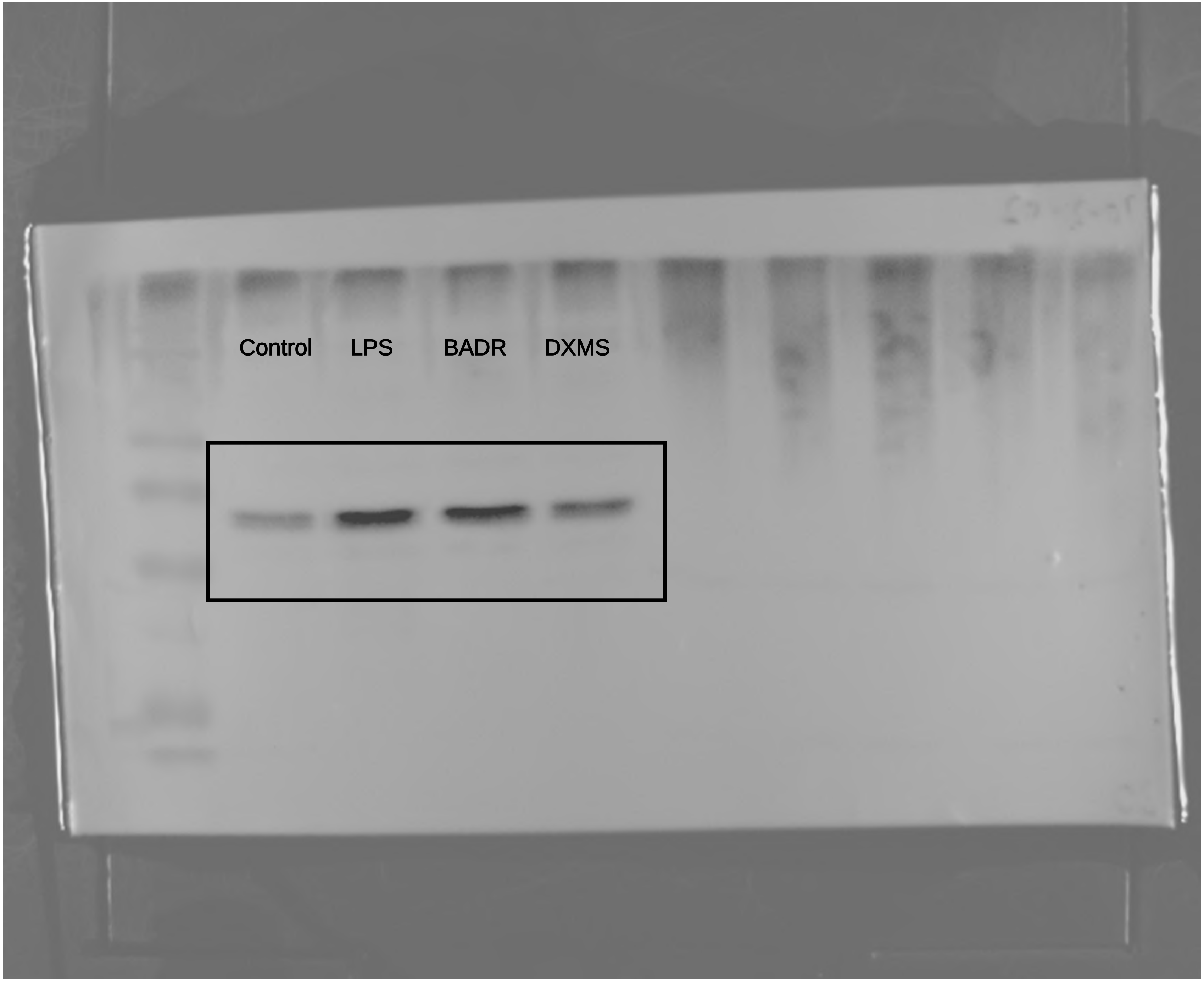

Figure 8B
STAT3
88 kDa

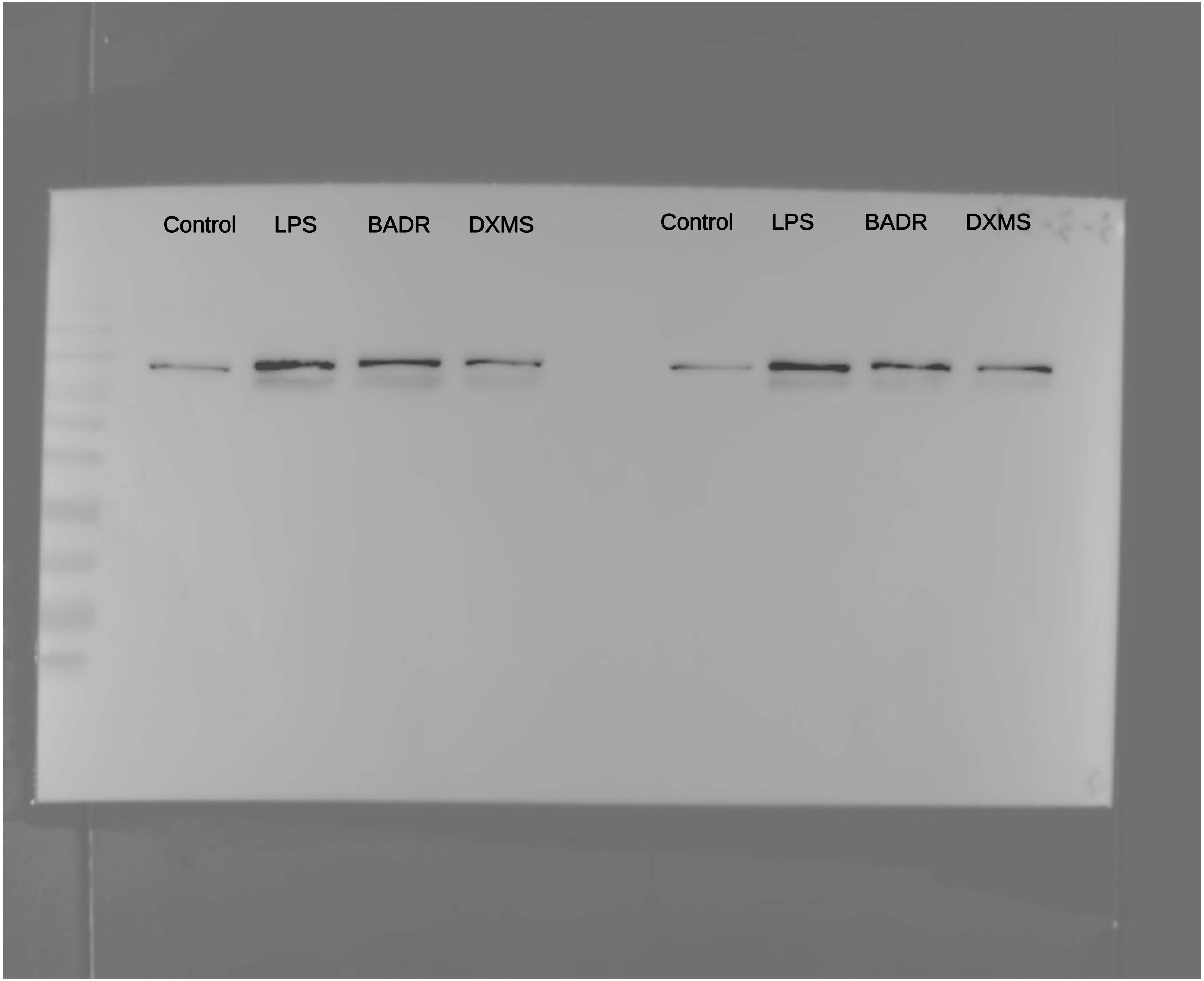

Figure 8B
STAT3
88 kDa

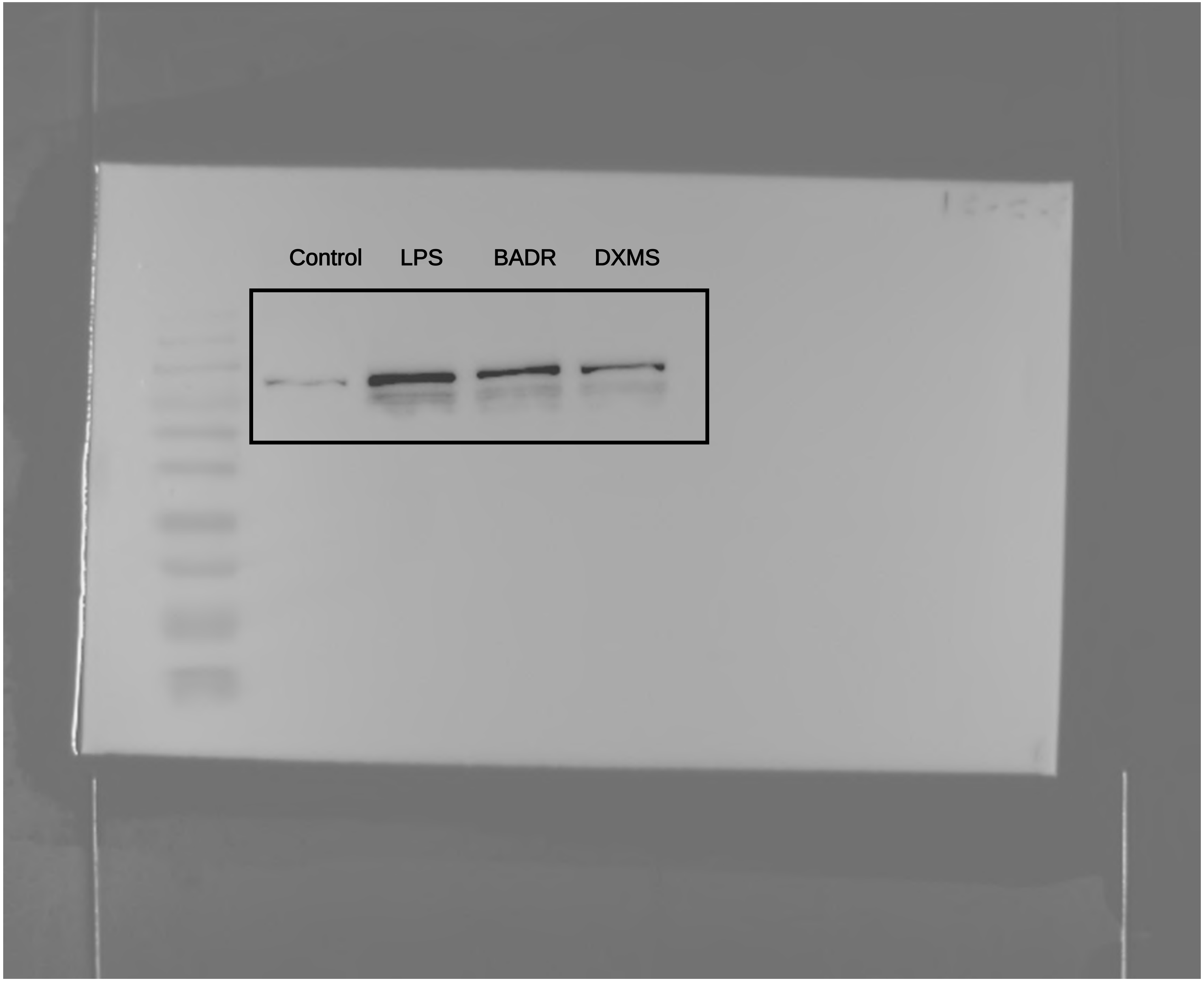

Figure 8B
BCL2
26 kDa

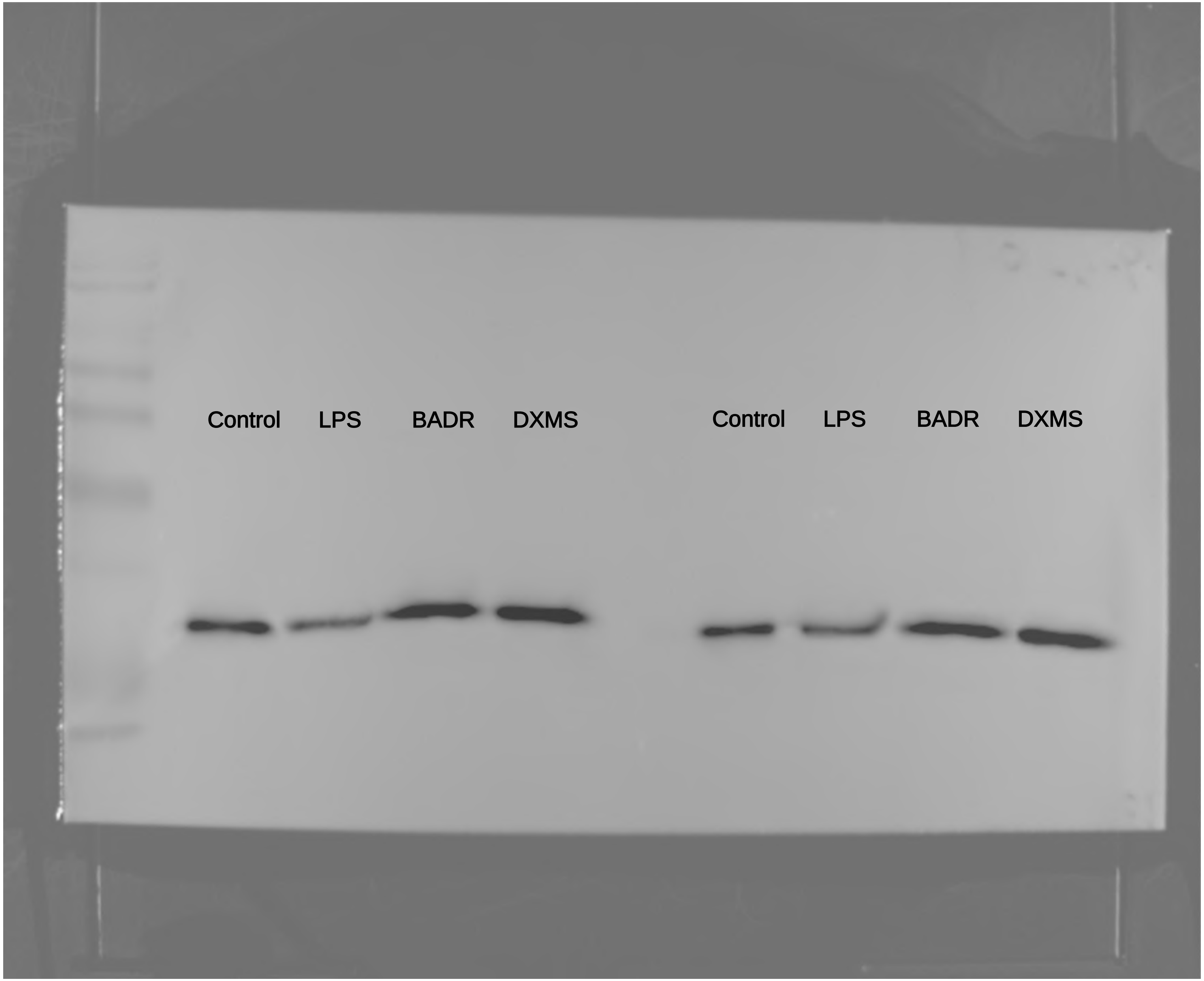

Figure 8B
BCL2
26 kDa

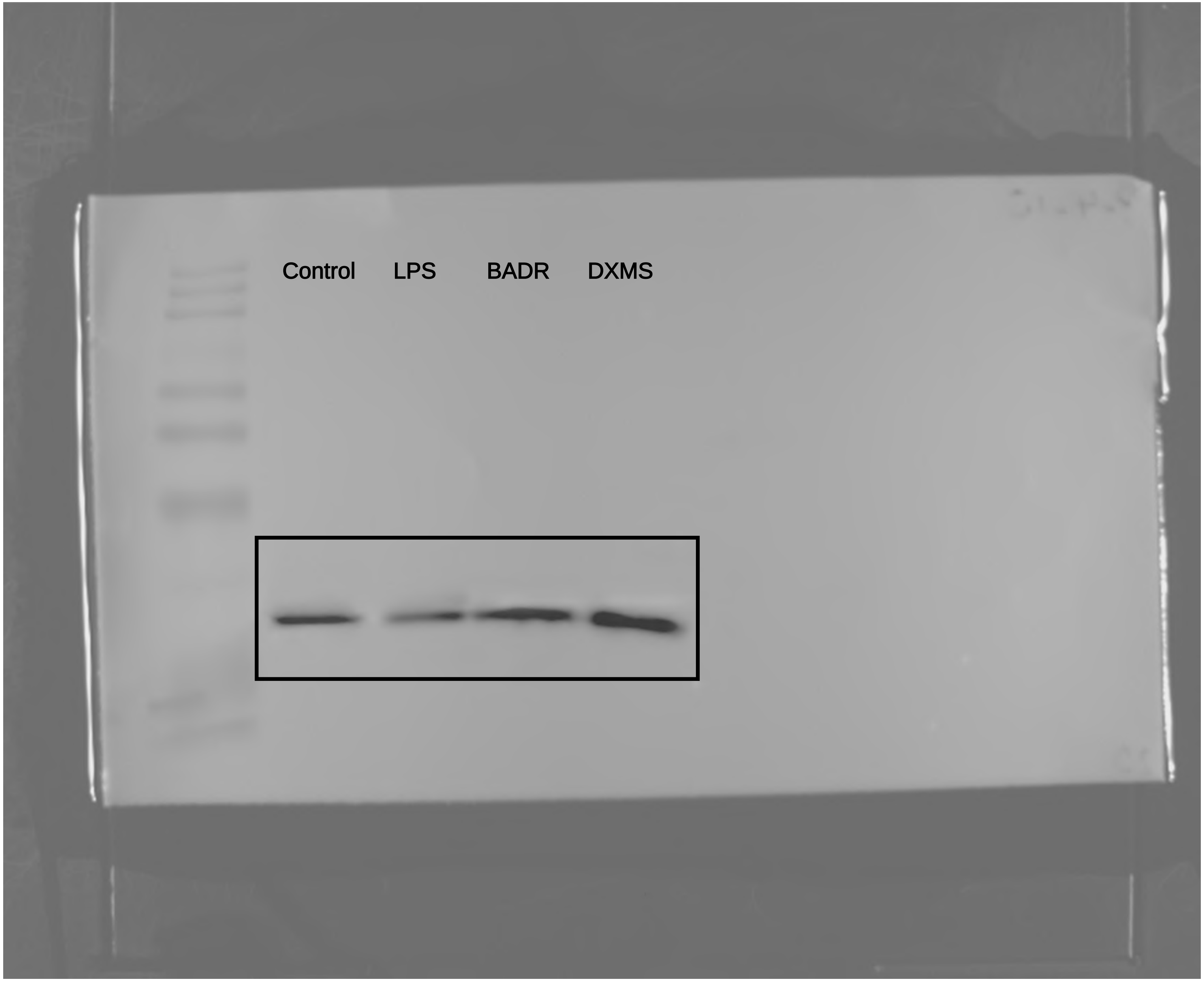

Figure 8B
β-Actin
42 kDa

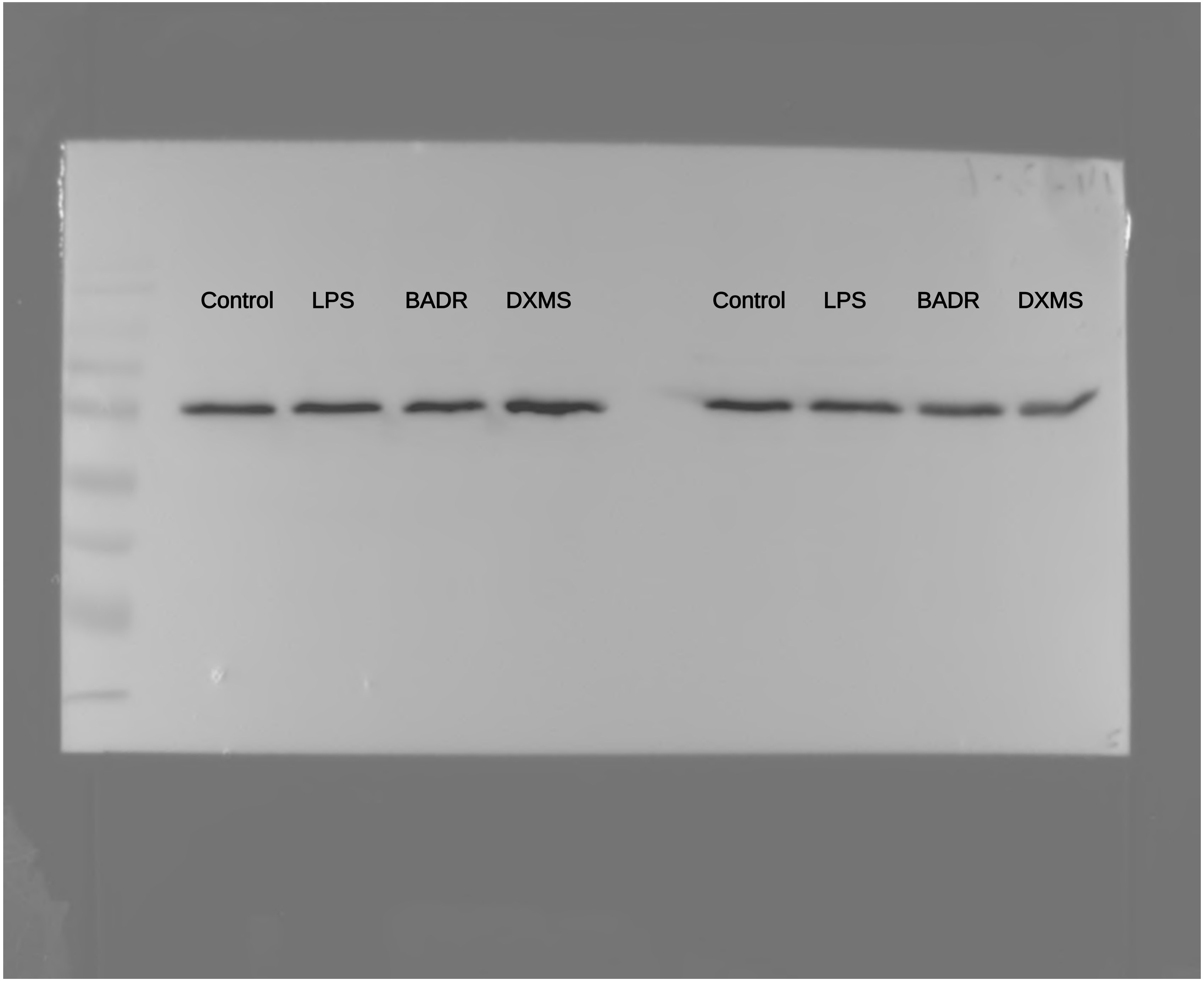

Figure 8B
β-Actin
42 kDa

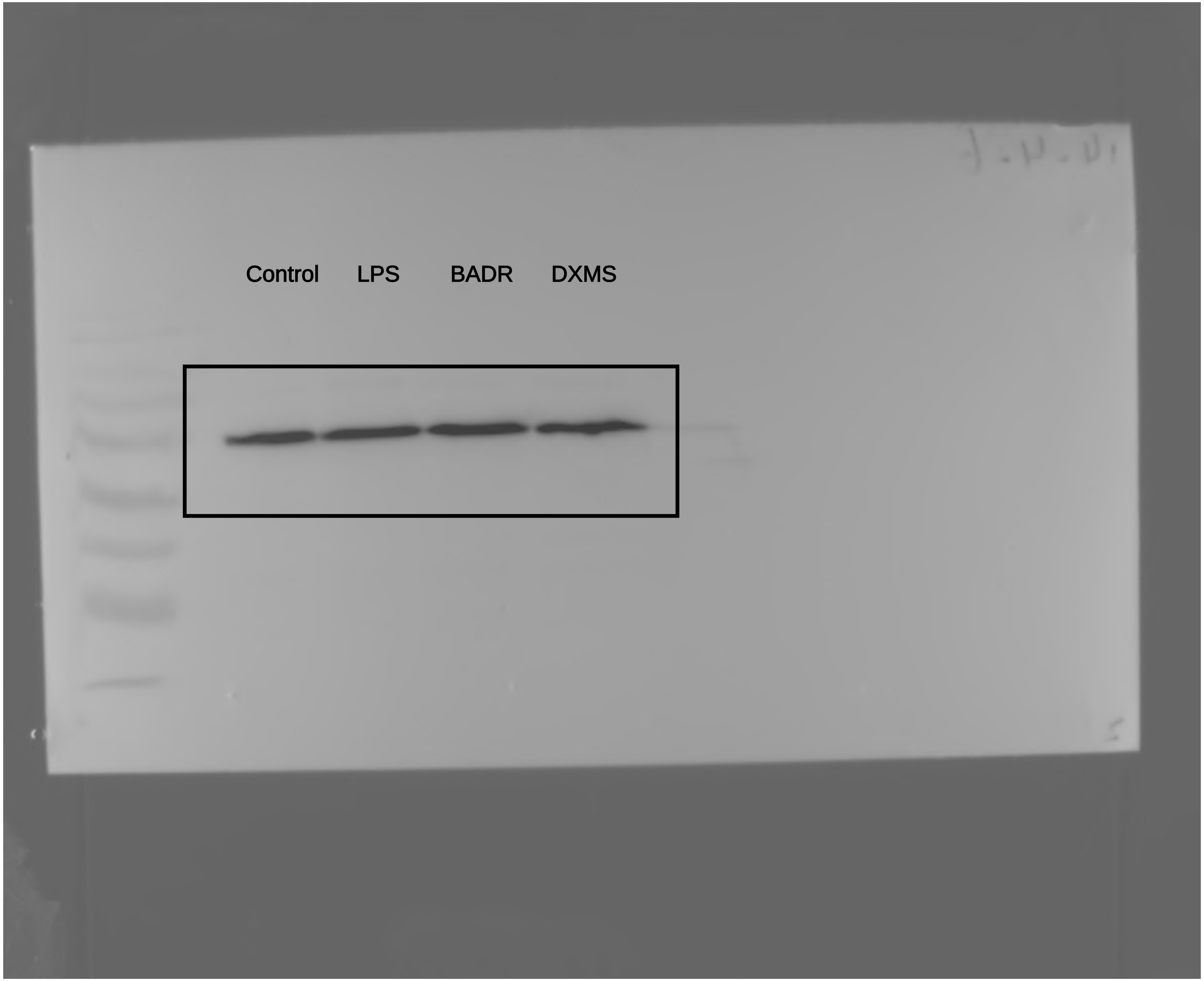

Figure 9A
EGFR
175 kDa

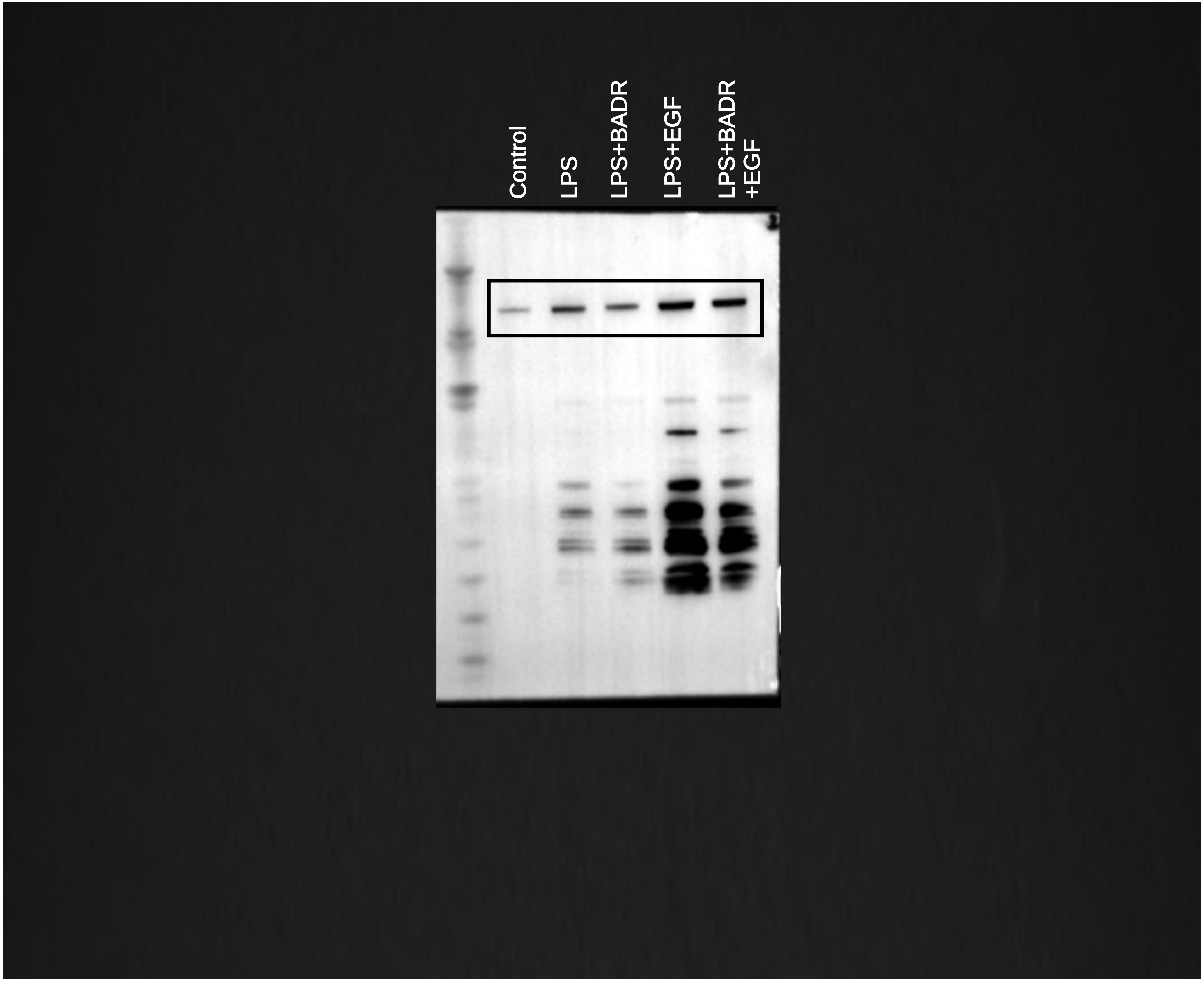

Figure 9A
EGFR
175 kDa

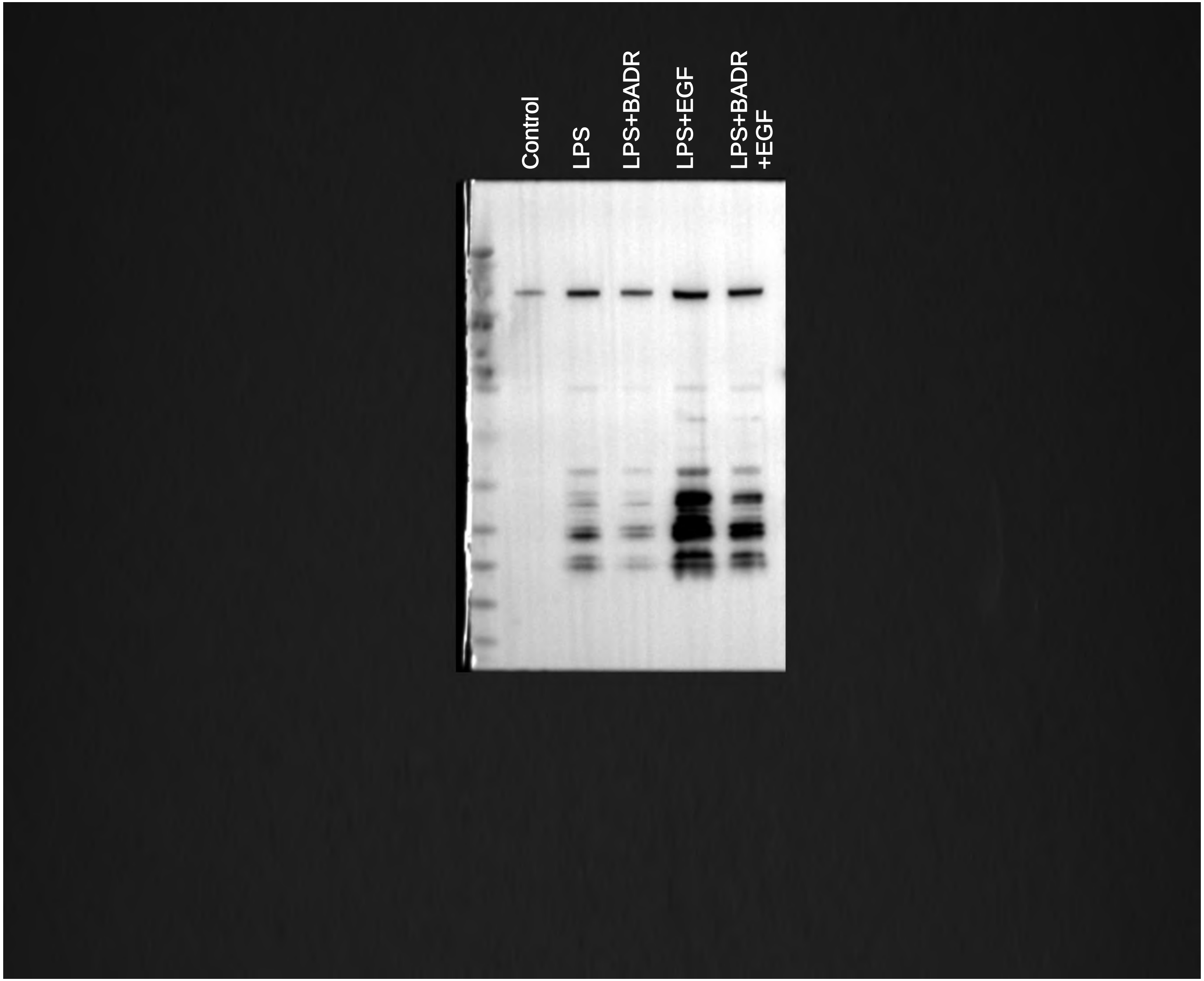

Figure 9A
EGFR
175 kDa

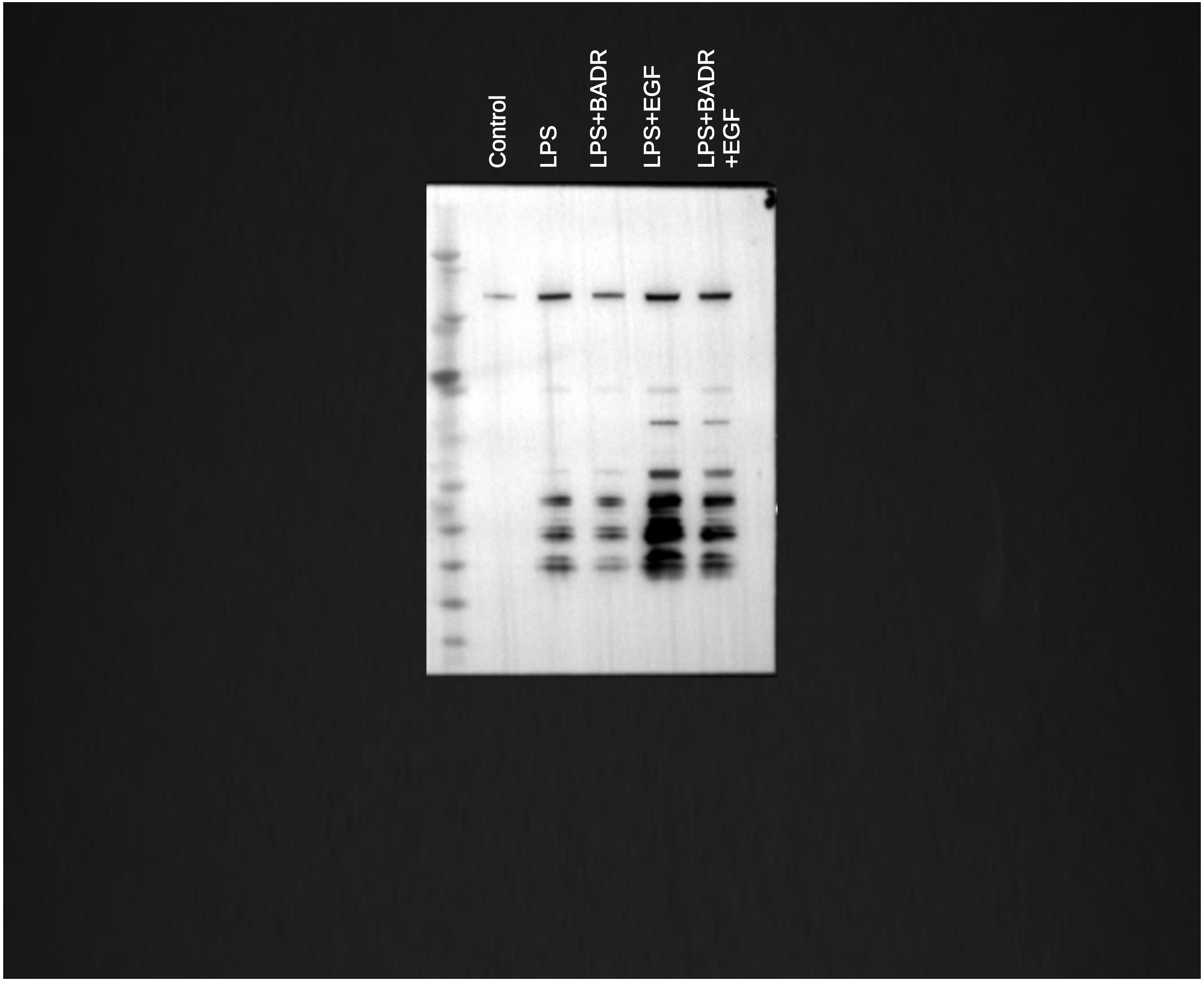

Figure 9A
p-EGFR
175 kDa

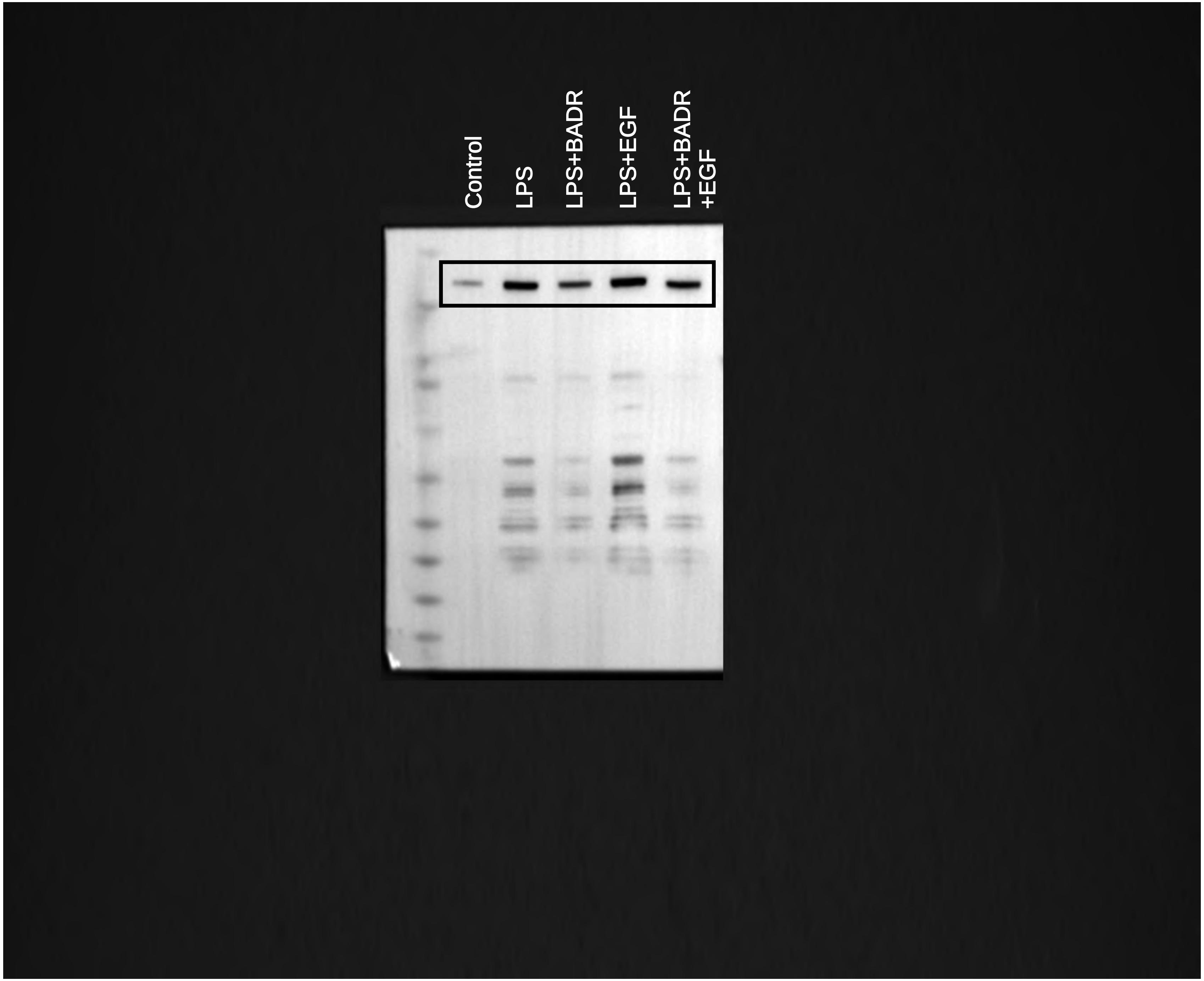

Figure 9A
p-EGFR
175 kDa

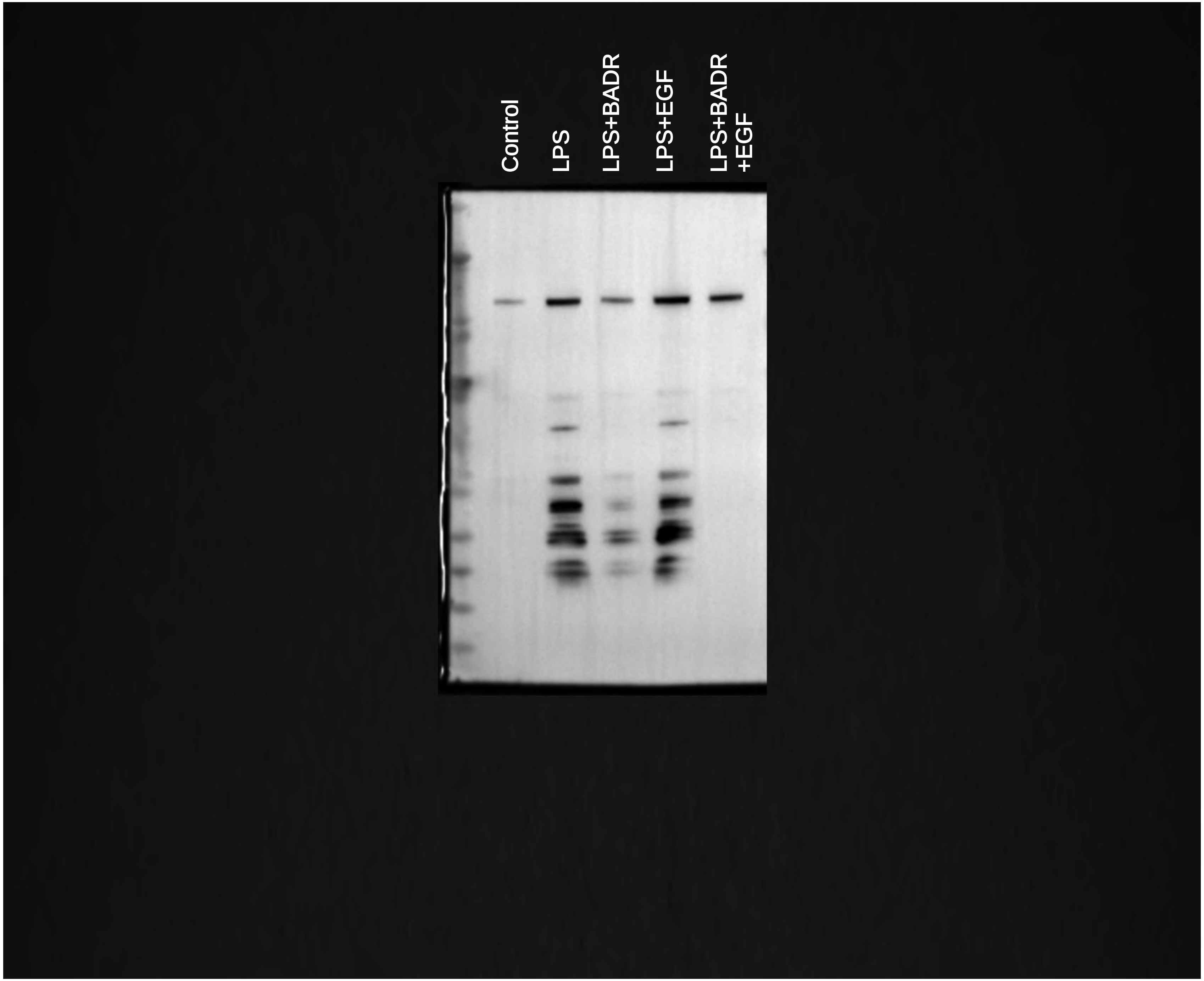

Figure 9A
p-EGFR
175 kDa

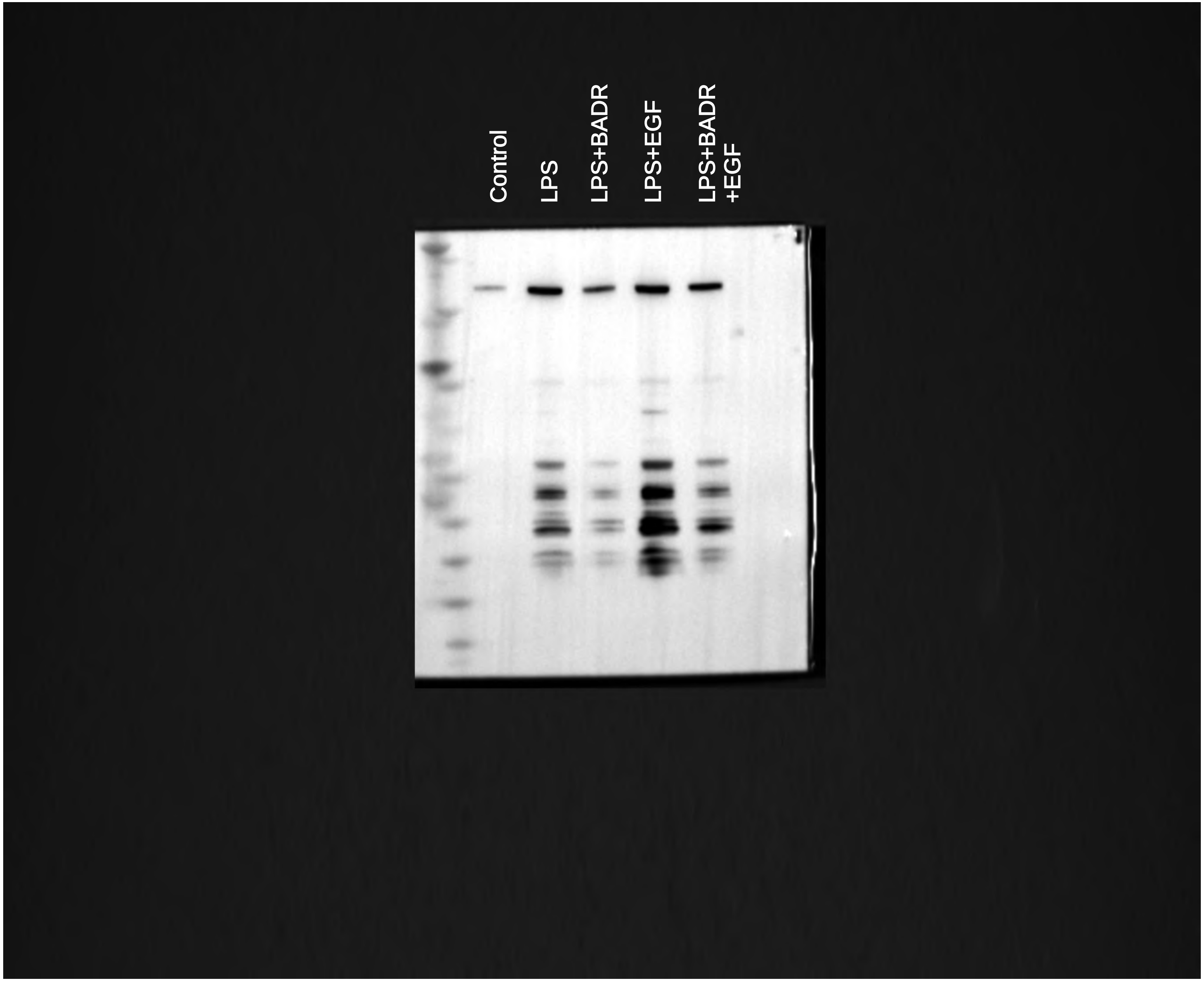

Figure 9A
STAT3
88 kDa

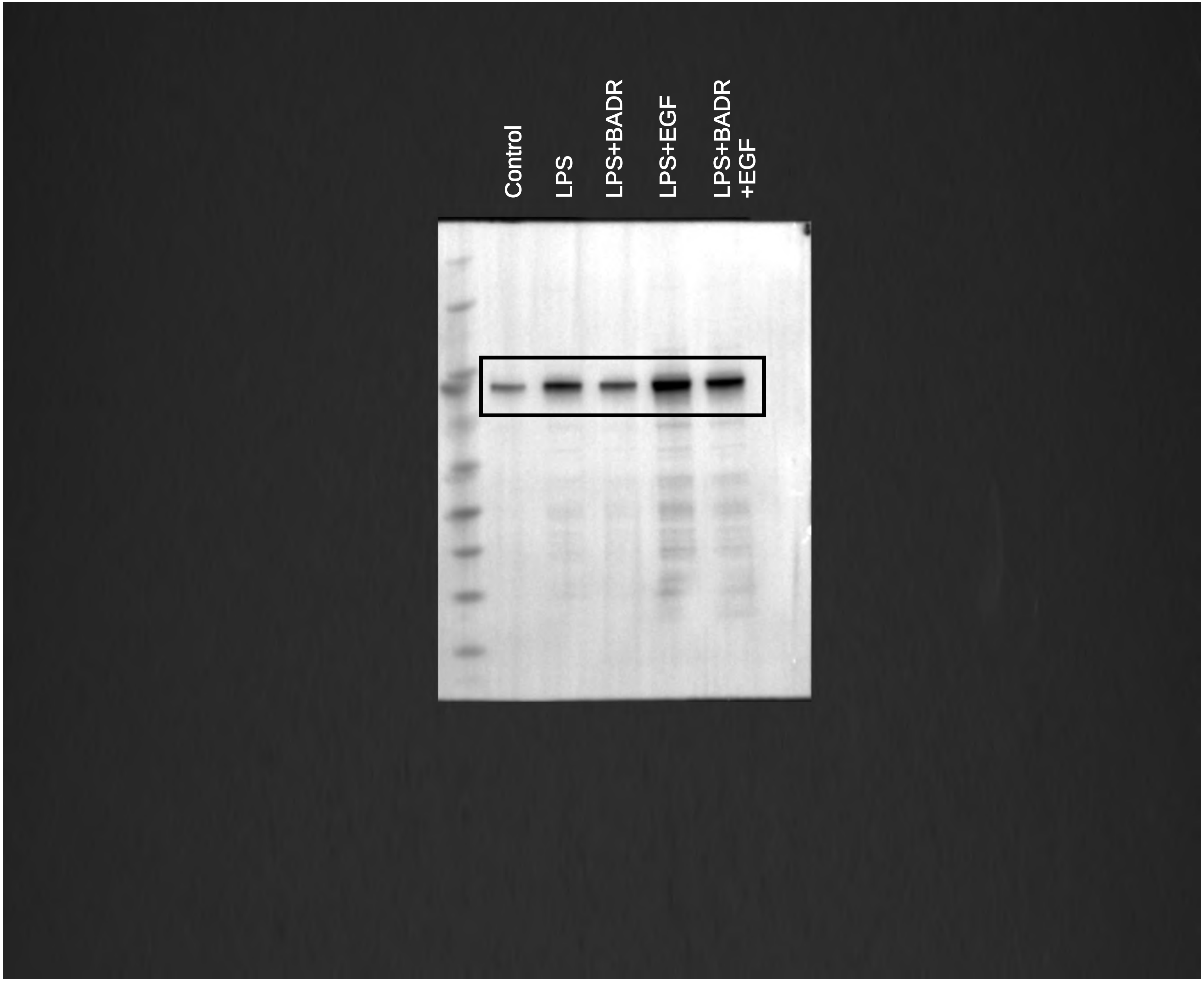

Figure 9A
STAT3
88 kDa

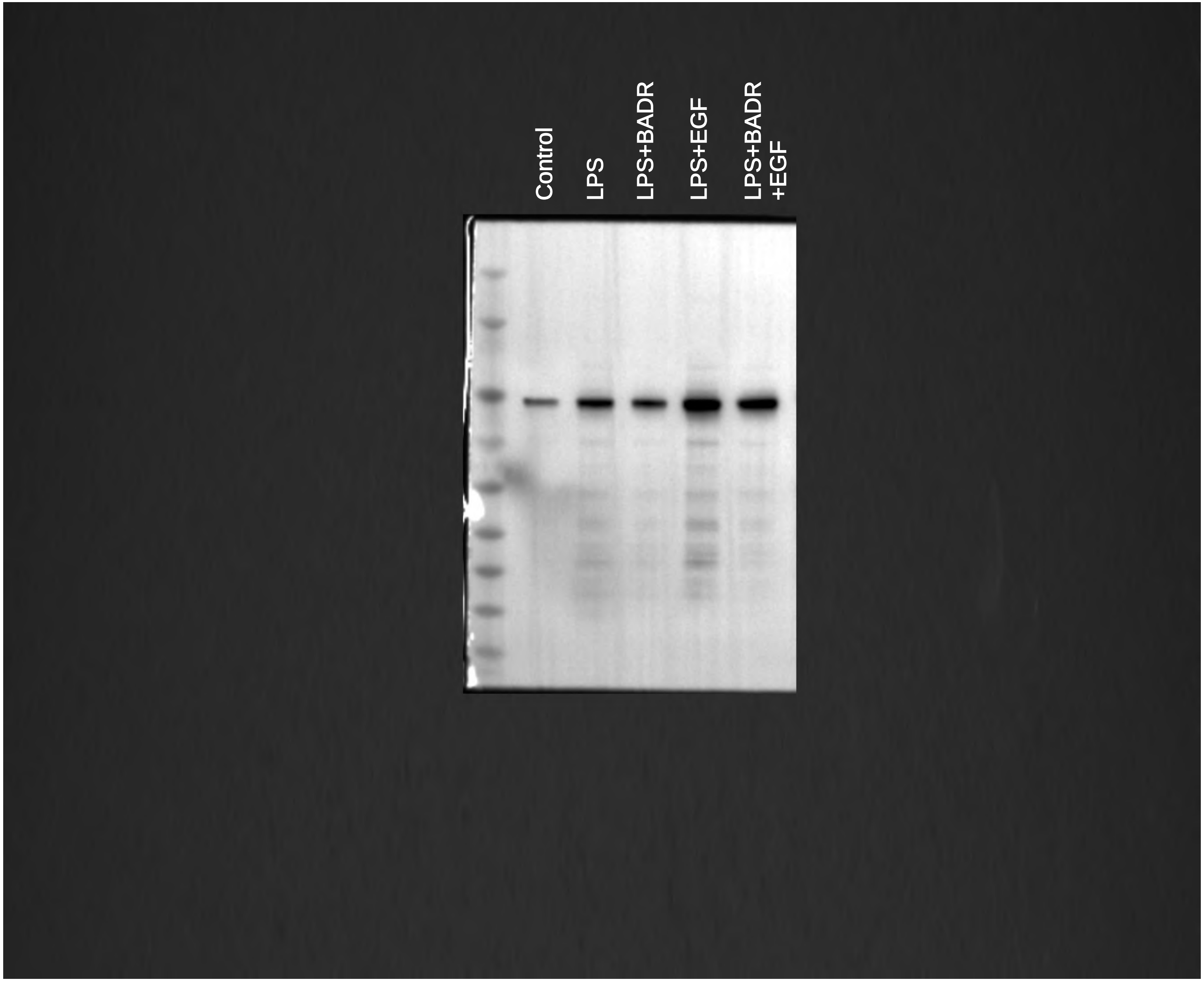

Figure 9A
STAT3
88 kDa

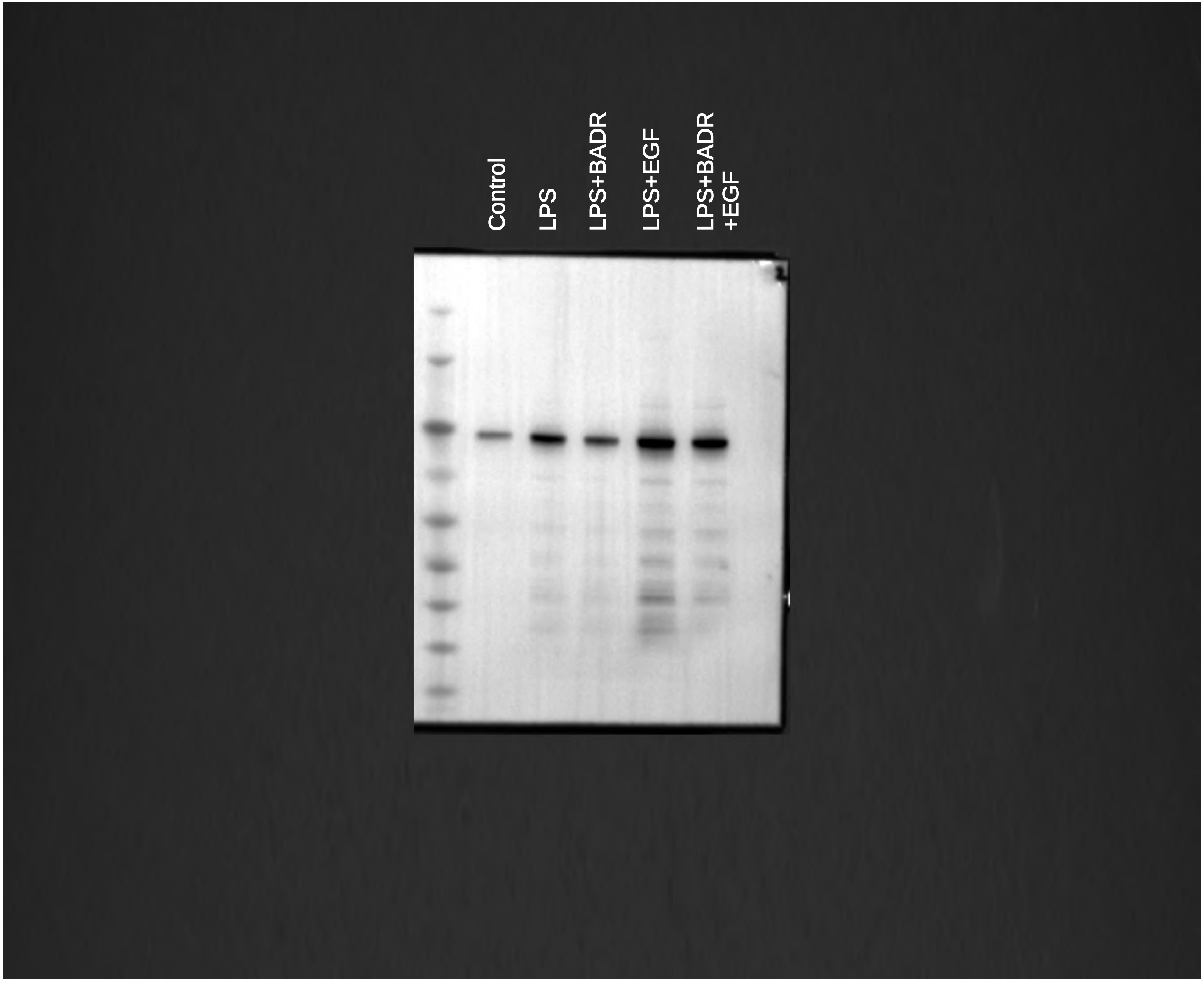

Figure 9A
p-STAT3
88 kDa

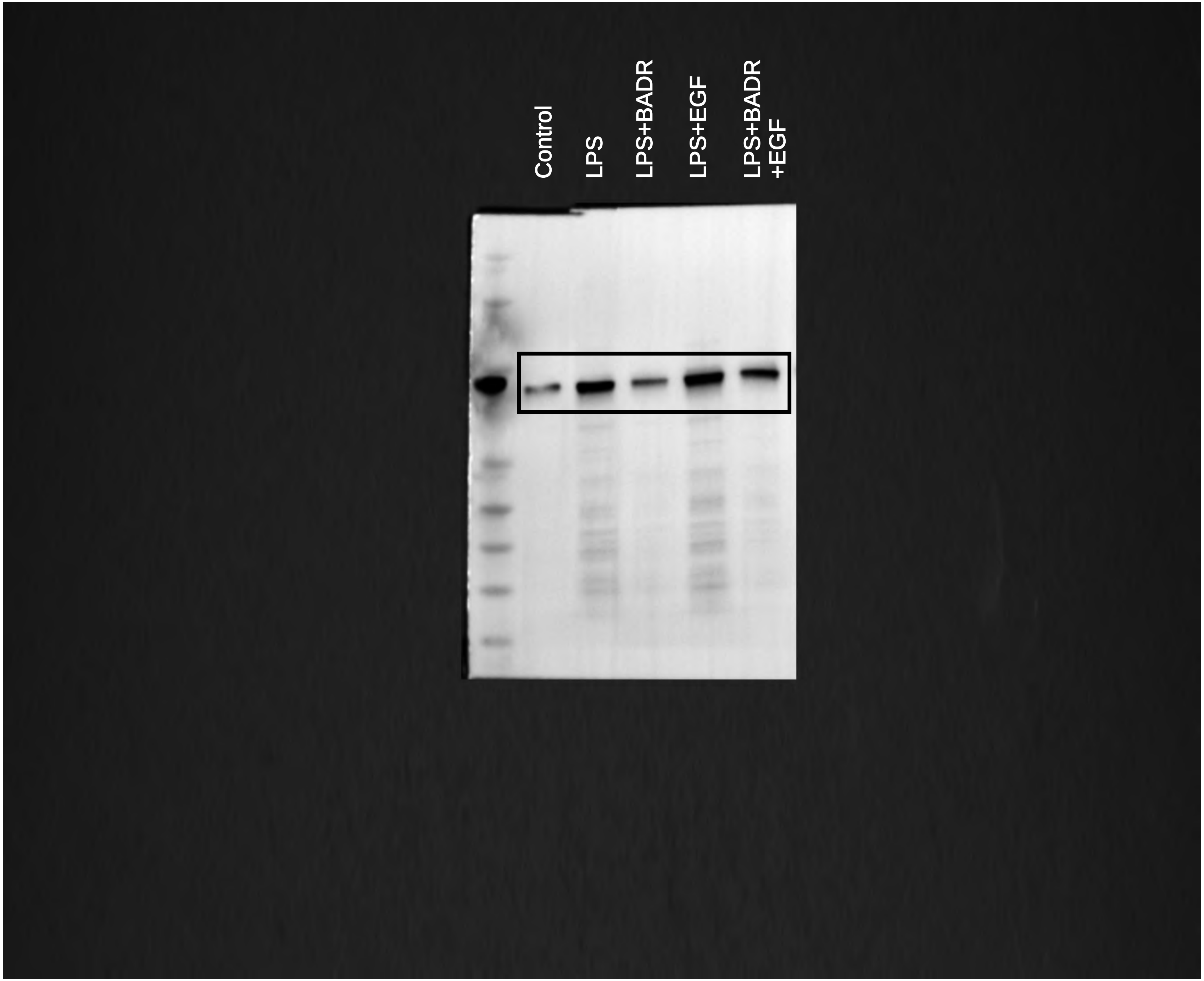

Figure 9A
p-STAT3
88 kDa

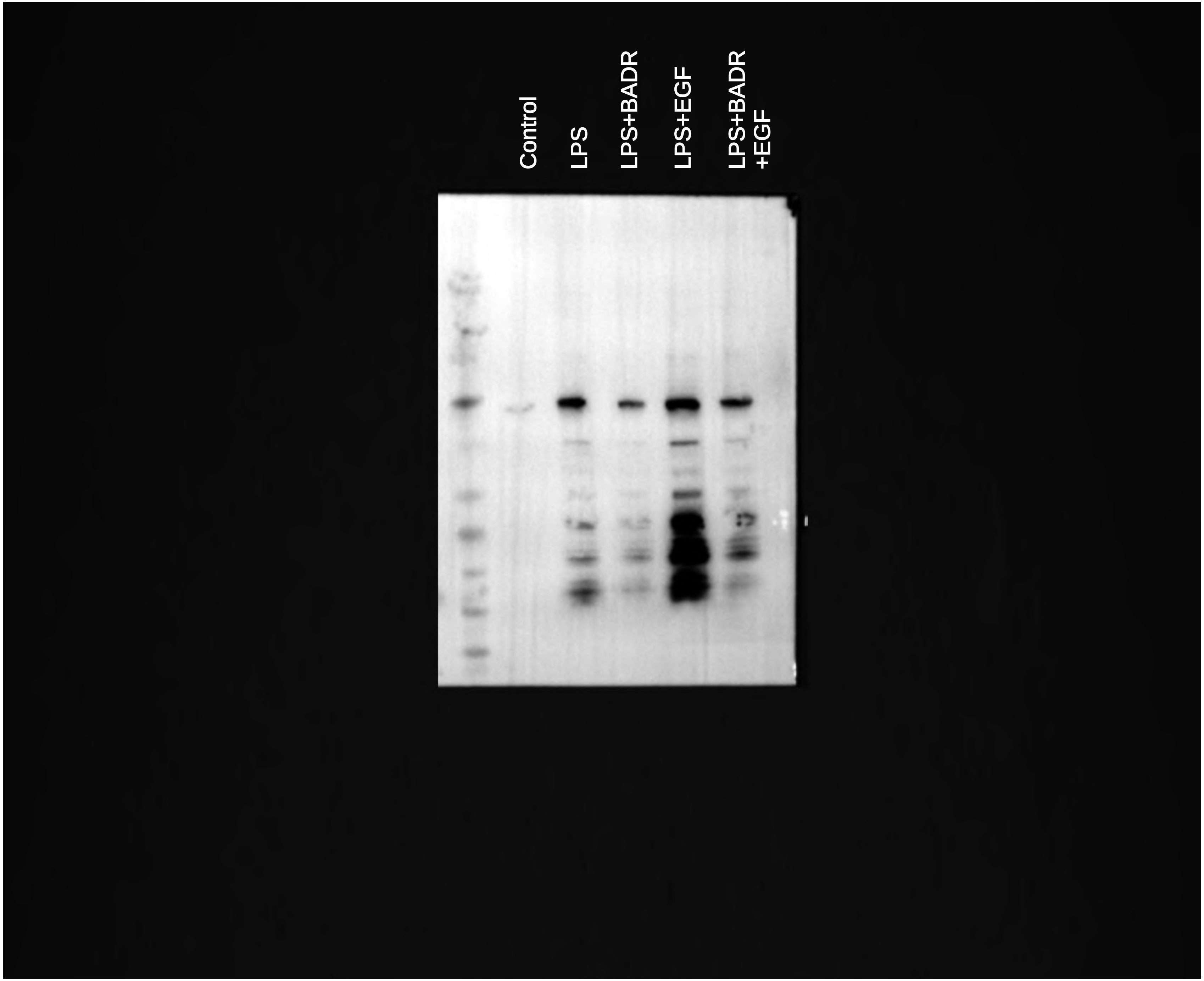

Figure 9A
p-STAT3
88 kDa

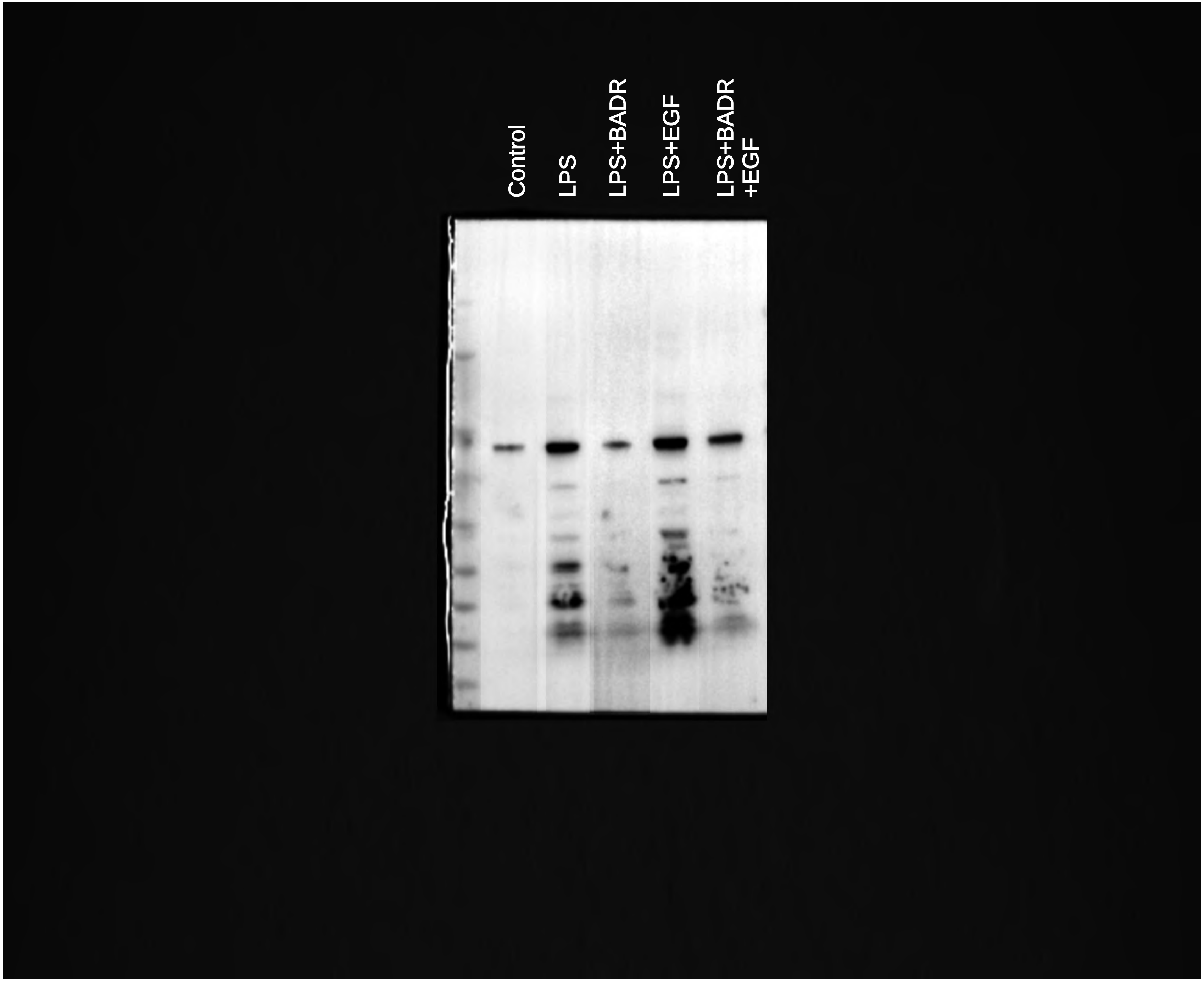

Figure 9A
β-Actin
42 kDa

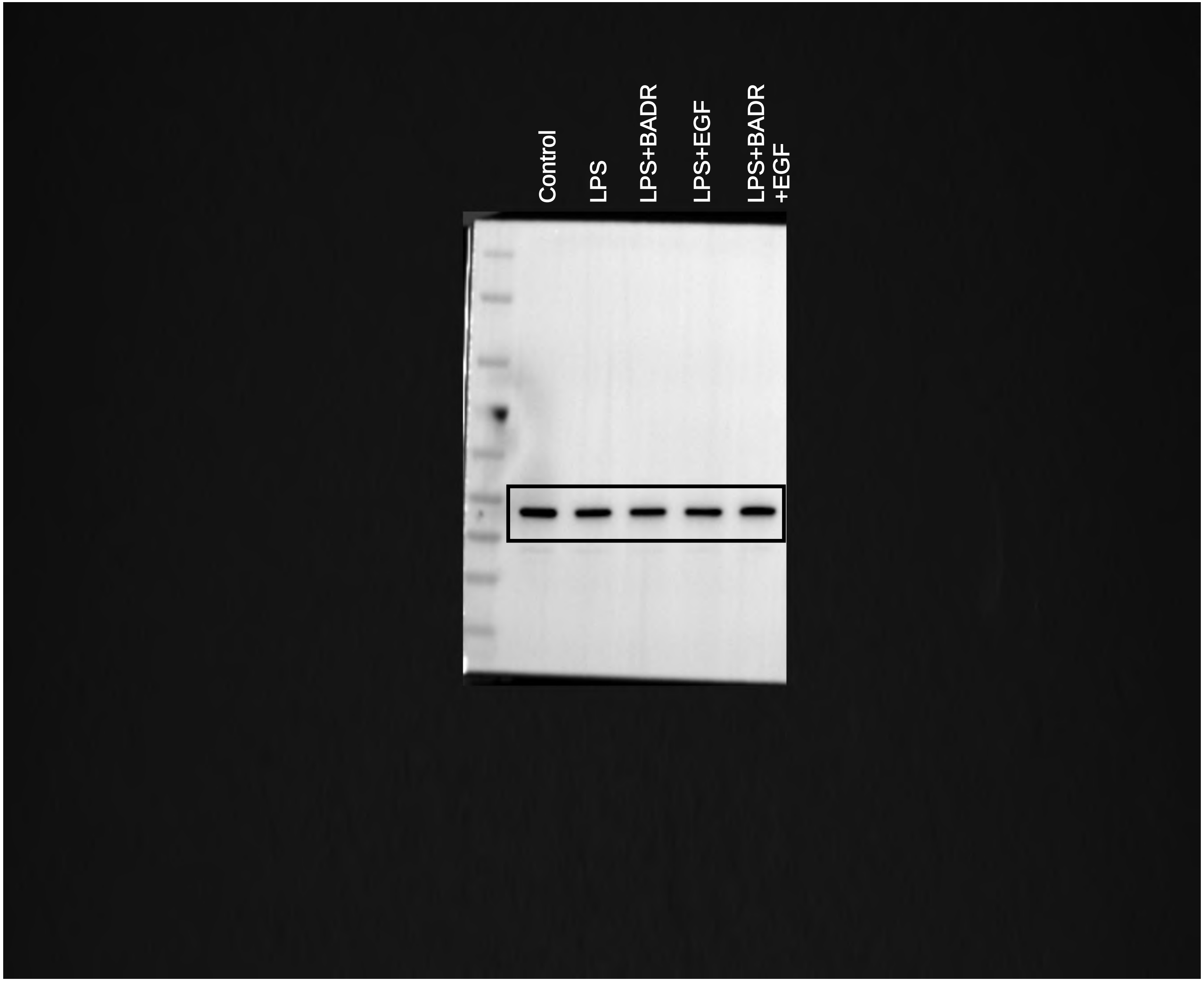

Figure 9A
β-Actin
42 kDa

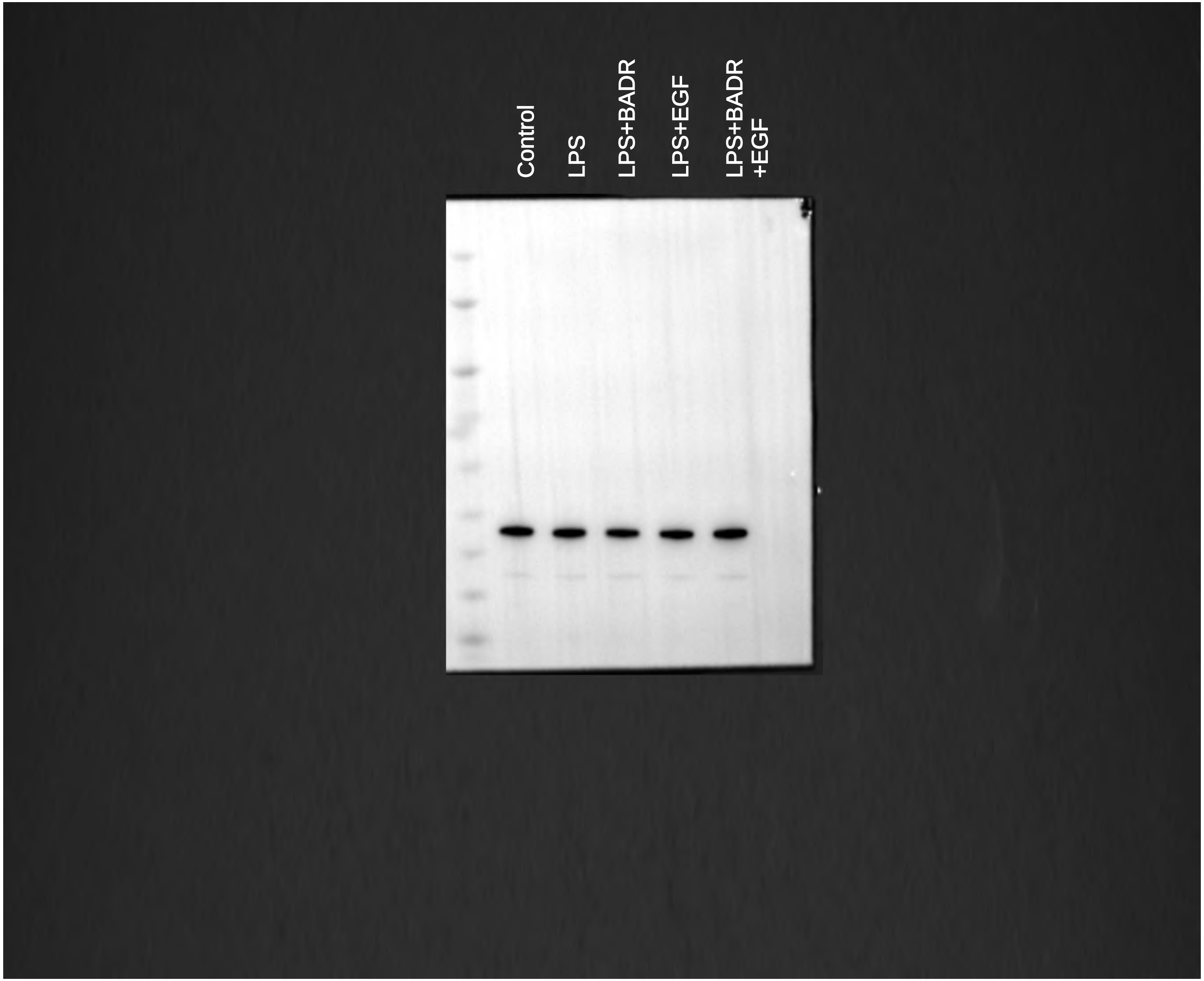

Figure 9A
β-Actin
42 kDa

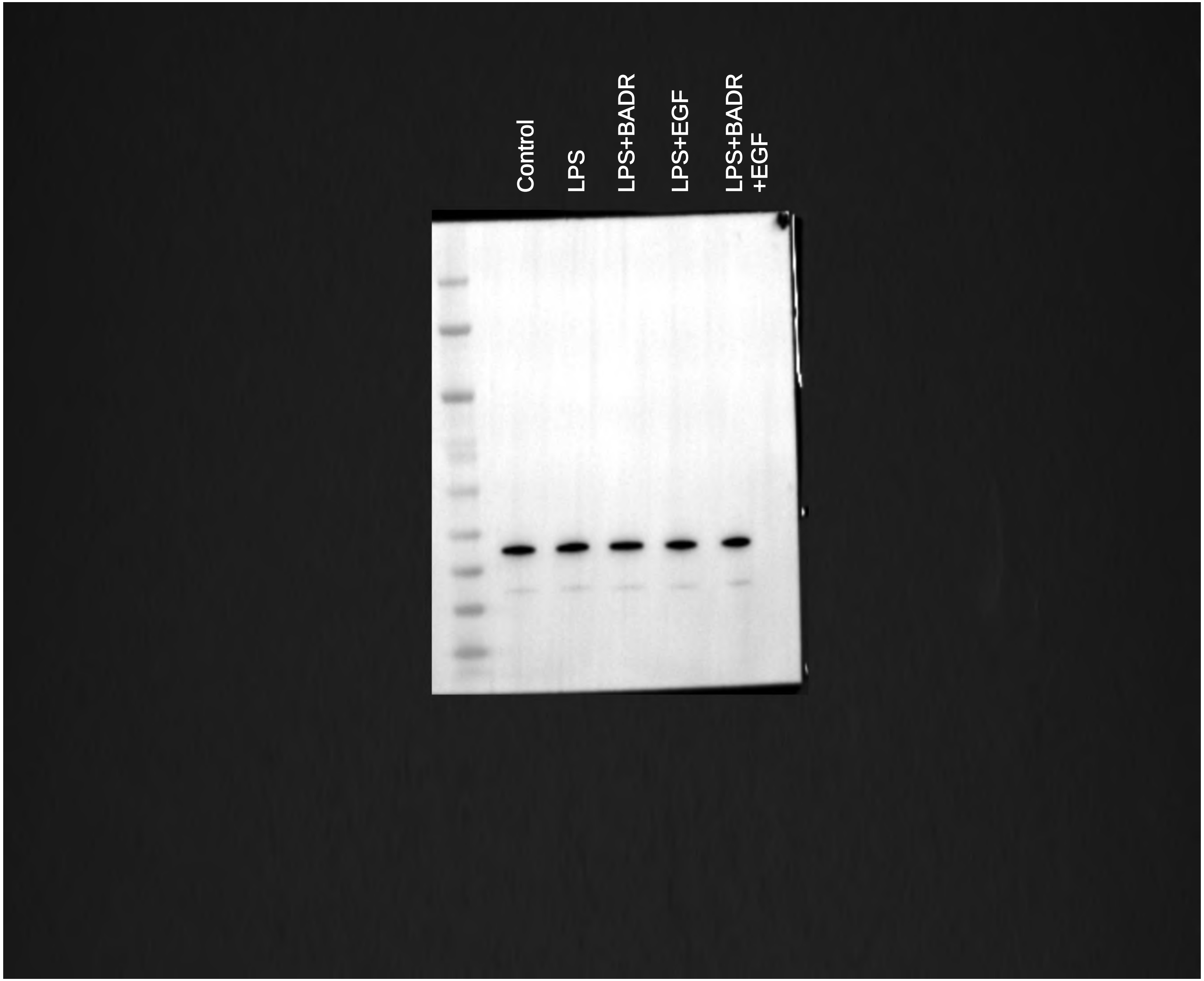

Supplement: Supplementary file 1 [file Data_Sheet_1.PDF]
